# The epidemiology of errors in data capture, management, and analysis: A scoping review of retracted articles and retraction notices in clinical and translational research

**DOI:** 10.1017/cts.2024.533

**Published:** 2024-05-17

**Authors:** Abigail S. Baldridge, Grace C. Bellinger, Oriana M. Fleming, Luke V. Rasmussen, Eric W. Whitley, Leah J. Welty

**Affiliations:** Feinberg School of Medicine, Northwestern University, Chicago, IL, USA

**Keywords:** Clinical and translational research, data management, data analysis, retractions, scoping review

## Abstract

**Introduction:**

To better understand and prevent research errors, we conducted a first-of-its-kind scoping review of clinical and translational research articles that were retracted because of problems in data capture, management, and/or analysis.

**Methods:**

The scoping review followed a preregistered protocol and used retraction notices from the Retraction Watch Database in relevant subject areas, excluding gross misconduct. Abstracts of original articles published between January 1, 2011 and January 31, 2020 were reviewed to determine if articles were related to clinical and translational research. We reviewed retraction notices and associated full texts to obtain information on who retracted the article, types of errors, authors, data types, study design, software, and data availability.

**Results:**

After reviewing 1,266 abstracts, we reviewed 884 associated retraction notices and 786 full-text articles. Authors initiated the retraction over half the time (58%). Nearly half of retraction notices (42%) described problems generating or acquiring data, and 28% described problems with preparing or analyzing data. Among the full texts that we reviewed: 77% were human research; 29% were animal research; and 6% were systematic reviews or meta-analyses. Most articles collected data *de novo* (77%), but only 5% described the methods used for data capture and management, and only 11% described data availability. Over one-third of articles (38%) did not specify the statistical software used.

**Conclusions:**

Authors may improve scientific research by reporting methods for data capture and statistical software. Journals, editors, and reviewers should advocate for this documentation. Journals may help the scientific record self-correct by requiring detailed, transparent retraction notices.

## Introduction

Retraction of inaccurate scientific articles is critical to ensuring the integrity of research and published literature. Incorrect findings, especially those that go unnoticed for months or years, may have broad repercussions in areas affecting human health, including clinical practice, drug discovery, and public policy. Reasons for retraction may be multifaceted, ranging from honest mistakes to egregious ethical and scientific misconduct [1].

The emergence of digitized publication databases in the 1980s and 1990s, such as PubMed, MEDLINE, and Embase, made possible the formal study of retractions. Chen and colleagues (2013) found that retractions in PubMed increased 8-fold from 2001 to 2011 [1]. An analysis of MEDLINE similarly found that retractions increased substantially, from 0.002% in the early 1980s to approximately 0.02% in 2005–2009 [2]. Recent studies across a range of disciplines have found that retractions may be increasing disproportionately to the number of articles published [3–5]. Growing interest in studying retractions and increased retraction volume has led to the creation of databases of retracted articles, such as the Retraction Watch Database (RWDB) [6].

Numerous articles have studied retractions within clinical areas, including cardiovascular medicine, radiology, surgery, nursing, cancer research, obstetrics, oncology, dentistry, and most recently, COVID-19 [3–5,7–13]. Many of these studies have analyzed the metadata from the RWDB or citation indices. More general investigations of retractions across biomedical sciences are often limited to article characteristics such as time to retraction, number of authors, country of origin, or reason for retraction as coded by the Retraction Watch (RW) team [14,15]. More granular investigation of the full text of the retracted articles may be prohibitively labor intensive; to date, most studies examining the full text tend to include fewer articles and, therefore, have limited generalizability.

Across fields and inquiries, research misconduct consistently emerges as a common reason for retraction [4,7,14,16–23]. The identification of research misconduct, its prevalence, and its prevention have received substantial attention [14,20,22,24]. In contrast, an estimated 21%–62% of retractions are related to unintentional errors [2,4,14,17,18,20,25]; the large range may be due in part to the difficulty of inferring authors’ intentions from retraction notices.

With the growth of team science and big data, the increased complexity of research may make preventable errors, such as those involving analytic methods, more likely to occur. To our knowledge, there has been no comprehensive study of articles in the biomedical literature that were retracted owing to mistakes in data capture, management, and/or analysis. This omission is critical: characterizing methodological and analytic mistakes is an essential step in improving their detection and prevention, thereby benefiting authors, reviewers, editors, and, ultimately, patient care, public policy, and human health.

We therefore conducted a first-of-its-kind scoping review of articles published from January 1, 2011 to January 31, 2020 that were subsequently retracted for reasons related to data capture, management, and/or analysis but not gross misconduct. Our scoping review builds on the existing literature in three important ways. First, we considered articles published in clinical and translational research, which includes a broad collection of articles across basic science, clinical medicine, and public health. Second, we extracted detailed information about methods, such as study design, how data were obtained, and statistical software, from the articles’ full text. Third, we reviewed retraction notices to categorize who initiated the retraction, author involvement, and high-level categories of the types of errors that occurred. Our review summarizes problems in the research pipeline related to the capture, management, and/or analysis of data so that authors, reviewers, editors, and publishers may consider steps to better detect and avoid these preventable errors.

## Methods

### Study design

The scoping review complied with Preferred Reporting Items for Systematic Reviews and Meta-Analysis extension for Scoping Reviews (PRISMA-ScR) guidelines and followed a preregistered protocol [26,27]. The corresponding PRISMA-ScR checklist is included in the supplemental materials (Online Supplement).

### Searches

The website RW was launched in 2010 as an initiative to assist the scientific community, and in 2014 became part of The Center for Scientific Integrity [28]. The RWDB, launched in 2018, is an index of retractions and at the time of data request was publicly available subject to a data use agreement [6]. The RWDB comprises a systematic and comprehensive compendium of retracted articles, including a detailed ontology to classify and describe the retracted articles. At the time of this writing, the RWDB included over 43,000 records and has been cited in over 140 research articles that aim to evaluate and understand trends, practices, and behaviors around retractions.

A total of 21,252 records were retrieved from the RWDB, current to March 12, 2020. Each record includes publication information (article title, journal, authors, publication date, DOI [digital object identifier], URL, PubMed ID), retraction information (date, retraction DOI, coded reasons), and coded subject lists (e.g., Business – Accounting, Neuroscience, History – Asia, Geology). Coded subjects and retraction reasons are applied to each retracted article by RW staff from prespecified banks of possible codes.

### Inclusion and exclusion criteria

Identification of the articles and retraction notices for inclusion was a four-step process: (1) data from the RWDB were reviewed to identify abstracts eligible for review; (2) abstracts were reviewed in duplicate to identify articles eligible for review; (3) we reviewed the retraction notices for all eligible articles; and (4) we reviewed articles if the full text could be located. Inclusion and exclusion criteria for each step are described below:

#### RWDB

- Year of Publication: RWDB records were subset to articles published on or after January 1, 2011. We chose this range to balance having a large sample of retracted articles with relatively recent articles.
- Subject Lists: We tabulated the frequency of each subject and reviewed the list for applicability (Supplemental Table 1). We first subsetted retraction records to include records associated with a subject of interest (e.g., Biology – Cancer, Neuroscience). Second, from this selection, we then excluded records for which the subject list contained terms unrelated to human subjects, medical or clinical research, or the practice of human subjects research (e.g., Foreign Aid, Astrophysics).
- Retraction Reasons: We tabulated the frequency of each retraction reason and reviewed the list for applicability (Supplemental Table 2). Retraction records were first subsetted to include records associated with a reason of interest (e.g., Concerns/Issues About Data, Error in Analyses). Second, we then excluded records for which the retraction reasons contained terms indicating gross misconduct (e.g., Falsification/Fabrication of Data, Ethical Violations by Author).

#### Abstracts

- English Language: Abstracts published in English were eligible.
- Human Subjects Research: Abstracts reporting research on human subjects were eligible.
- Clinical and Translational Research: For abstracts that did not explicitly report human subjects research, we determined if they were reporting clinical and translational research following guidelines published by the National Institutes of Health. We excluded abstracts that reported “basic research,” but we did include abstracts that reported “preclinical research,” defined as connecting “basic science of disease with human medicine [29].”

#### Retraction notices

- Retraction notices matching the DOI in the RWDB were reviewed for all eligible articles.

#### Full-text articles

- Eligible articles were reviewed if the full-text article matching the DOI in the retraction notice could be accessed by the study team using journal subscriptions available through Northwestern University’s library system.

### Data collection and management

The Research Electronic Data Capture (REDCap) tool hosted at Northwestern University was used throughout the review processes for data entry and importing article information from the RWDB (Online Supplement) [30].

#### Abstracts

Each abstract was located by searching for the DOI or article title as recorded in the RWDB. Abstracts were located and assessed for inclusion in duplicate by two independent reviewers (ASB, LVR, EWW, or LJW; Online Supplement). Conflicting decisions were resolved through review by a third team member, followed by discussion with all four reviewers.

#### Retraction notices

The Committee of Publication Ethics (COPE) guidelines were used to inform a framework for qualitative review of the retraction notices (Online Supplement) [31]. Information on the involvement of authors, editors, journals, and publishers in the retraction process was extracted by a single reviewer (ASB, GCB, OMF, LVR, or LJW). The retraction notices were examined in duplicate by two independent reviewers (ASB, GCB, OMF, LVR, or LJW) to qualitatively code if the underlying reasons for retraction were related to either (1) generating or acquiring data and/or (2) preparing or analyzing data, defined below.

- *Generating or acquiring data*: Examples include laboratory error, sample contamination, incorrect articles included in a meta-analysis, wrong cell types, incorrect patient identification for a case, incorrect data pulled from an electronic health record or other system, misinterpretation of diagnoses or tests in the data pull, unreliable data or concerns about data, error in data, or loss of data. This category also includes instances for which investigators regenerated data that were inconsistent with the original data, or if there was a problem with data storage (i.e., acquiring, saving, and retaining).
- *Preparing or analyzing data*: Examples include data preparation, data cleaning, data normalization, unit conversion, incorrect data merge, variable coding, statistical analysis, or incorrect standard errors. This category also includes instances for which concerns were noted about results, as long as the wording suggested that concerns were related to data analysis rather than benchtop work or data generation.

When retraction notices did not map to either of the coded categories, any other reasons for retraction were excerpted into an “Other” category.

- *“Other” reasons*: Examples include general statements about “could not be replicated” that do not refer specifically to data or results, questions about the integrity of the data (not about the process generating the data), and duplicate figures or articles published in another context.

Conflicting decisions were resolved through consensus review.

#### Full-text articles

A single team member (ASB, GCB, OMF, LVR, or LJW) searched by DOI or article title, reviewed the retrieved article to ensure that it was the version that was retracted, and uploaded the article to the REDCap database. The team member then extracted the following information (Online Supplement):

- Authorship: The number of authors and their contributions.
- Data: Whether data were collected *de novo* or previously collected, how data were captured and stored, and if data were available publicly or available upon request.
- Study Design: Whether the study was a systematic review or meta-analysis, animal study, and/or human subject research; the specific study design for human subjects research.
- Methods and Analysis: If a statistical analysis plan was prespecified; what software was used for data analysis; and any other information about reproducible research (e.g., availability of code or software).

Data extraction was restricted to information contained within the full text or the supplemental materials available with the original publication. A random sample (*n* = 44, 6%) of full-text articles were reviewed and data were extracted in duplicate (LVR and LJW). Discordant data from this verification process were evaluated by all reviewers.

### Statistical analyses

Initial data management and application of inclusion and exclusion criteria on raw data from the RWDB were performed using SAS v9.4 (SAS Inc., Cary, NC). All other analyses were performed using Stata v17 (StataCorp LLC., College Station, TX) or R v4.0.1 (https://www.R-project.org/). The manuscript was prepared using StatTag [32]. All continuous variables are summarized with medians and interquartile ranges. Categorical variables such as article characteristics and retraction reasons are summarized with frequencies and percentages. We used Kappa statistics to summarize concordance for data collected in duplicate: whether or not abstracts described clinical and translational research; retraction reasons being related to getting or acquiring data or preparing or analyzing data; and data extracted from a random subset of *n* = 44 full-text articles. We reviewed the Kappa statistics for patterns based on reviewer dyads or article characteristics.

## Results

### Eligible publications and reviewer agreement

**Figure 1.**
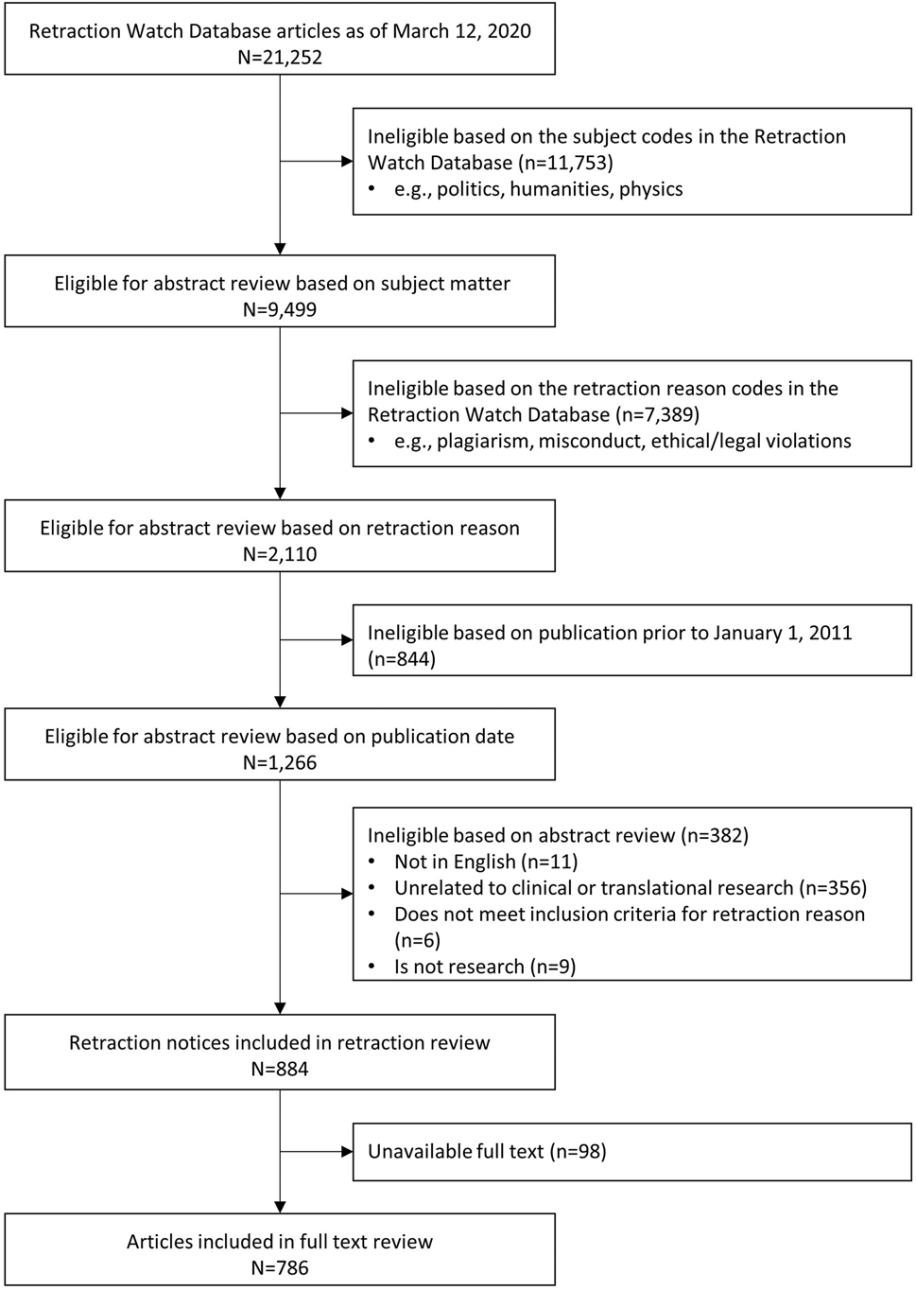
Flow chart of Retraction Watch Database records, eligible abstracts, eligible full texts, and complete reviews.

Of 21,252 records retrieved from the RWDB, 1,266 (6%) were eligible for abstract review, and 884 (70%) of these abstracts were in English and related to clinical and translational research (Fig. 1). All 884 retraction notices were reviewed in duplicate for retraction reason. Of the 884 eligible abstracts, 786 (89%) had full article text available online through Northwestern University’s library system.

Agreement during the entire review process ranged from moderate to almost perfect. During abstract review, there was substantial agreement about what constituted “clinical and translational research” (*κ* = 0.66). For the 170 abstracts (13% of 1,266) for which the two initial reviewers disagreed about “clinical and translational research,” the team reviewed and resolved differences by consensus. When coding retraction reasons from retraction notices, there was moderate agreement regarding whether the retraction was related to generating or acquiring data (*κ* = 0.50), and substantial agreement regarding whether the retraction was related to preparing or analyzing the data (*κ* = 0.67). The team reviewed the 281 (32% of 884) retraction notices for which there were disagreements regarding retraction reason and resolved differences by consensus. Among the random sample (*n* = 44 [6%]) of full-text articles for which data were extracted in duplicate, concordance of the blinded reviewers had substantial agreement (highest *κ* = 0.97; lowest *κ* = 0.73; Supplemental Table 3). There was some variability in the ability to locate articles based on DOI; 3 of the 44 articles (7%) were found by one reviewer but not by the other.

### Characteristics of retraction notices

Among 884 retractions, the median (interquartile range) time from publication to retraction was 1.0 years (0.4–2.4). The median (interquartile range) word count of the retraction notices was 109 (70–169) (Table 1).

**Table 1.** Characteristics of 884 retraction notices in clinical and translational research

Characteristic	*N*	(%)
Time from publication to retraction, years, median (IQR)	1.0	(0.4–2.4)
Word count of retraction notice, median (IQR)	109	(70–169)
**Entity(ies) named in the retraction notice as retracting the article** ^1^		
Author(s)	512	(58%)
Ambiguous Editor(s)/Journal/Publisher^2^	29	(3%)
Editor(s)	381	(43%)
Journal	45	(5%)
Publisher	139	(16%)
Other^3^	22	(2%)
Not Stated	53	(6%)
**Entity that initiated the investigation or discovered the retraction should occur**		
Author(s)	358	(40%)
Editor(s)/Journal/Publisher	29	(3%)
Letter to Editor	13	(1%)
External Investigation	41	(5%)
Readers	52	(6%)
Unnamed Entity	95	(11%)
Other	33	(4%)
Not Stated	277	(31%)
**Author involvement in the retraction process**		
Yes, and no authors explicitly disagreed with retraction	697	(79%)
Yes, and some author(s) explicitly disagreed with retraction	65	(7%)
Author(s) unresponsive	17	(2%)
Not stated	105	(12%)
**Types of Errors**		
Getting or Acquiring Only	336	(38%)
Preparing or Analyzing Only	217	(25%)
Getting or Acquiring and Preparing or Analyzing	31	(4%)
Something Else	294	(33%)
Unknown	6	(1%)

1 Response categories were not mutually exclusive.

2 e.g., “We retract this article…”

3 e.g., institutions, medical centers, professional organizations.

#### Entities involved in the retraction

Among 884 notices, author involvement in the retraction process was common. For most retractions, authors were named as the entity retracting the article (*n* = 512, 58%). Authors were also the most common entity to initiate the retraction process (*n* = 358, 40%). Authors were usually involved in the retraction and did not explicitly disagree with retracting the article (*n* = 697, 79%), but in 7% of retractions (*n* = 65), authors were involved and at least one disagreed. Of note, 12% of retraction notices (*n* = 105) did not address author involvement.

The next most common entities named in retracting the article were editors (*n* = 381, 43%) and publishers (*n* = 139, 16%). Several journals used a standard format for their retraction notices that consistently listed all three – authors, editors, and publishers – as formally retracting the original article. Nearly one-third of retraction notices did not state who initiated the retraction (*n* = 277, 31%).

#### Types of errors

Among 884 retraction notices, 42% (*n* = 367) described problems with generating or acquiring data, and 28% (*n* = 248) described problems with preparing or analyzing data. Both types of errors were described in 31 notices (4%) (included in the percentages above). Only 6 retraction notices contained insufficient information to make any determination about the retraction reason (e.g., “this article has been withdrawn”) [33]; another 294 described reasons that were unrelated to our categories, such as problems with original study design or conduct, or that were too vague to classify (*“wrong content with serious consequences;” “serious scientific errors”*) [34,35]. Table 2 provides illustrative excerpts of types of errors.

**Table 2.** Selected extracts of retraction notices by type of error

**Problems with Getting or Acquiring Data**
“…there may have been some fluorescence impurity/contamination with cAMP when they conducted their original experiments.” [79]
“…the authors discovered after publication that one of the cell lines described in the article had been unintentionally misidentified” [37]
“… we discovered a technical error in the measurement of CF sputum phenazines” [36]
“… incorrect cohort identification (ie, we missed many patients who were eligible for cardiac rehabilitation). We did not query all relevant codes used to identify the cohort of patients with ischemic heart disease.” [39]
“…more than 500 cases of the total 1882 cases of hernia patients presented in the paper were actually hydrocele of tunica vaginalis, not hernia” [80]
“transgenic mice reported in Supplementary Figs. 3 and 6 and in Figs. 4 and 5 were misidentified” [38]
“… the data collected from self-report of the HBV vaccination remains unverified, and potentially subject to errors.” [81]
“…a number of subject data points had been mistakenly duplicated …” [82]
“… we identified incorrectly entered data for six subjects on two variables.” [40]
“…some data points that should have been entered as a positive result were instead entered as having a negative result…” [41]
“…data that was input in SPSS is from another questionnaire” [83]
“…underlying data for the reported experiments are unavailable due to issues including the amount of time that has passed (seven years)…” [84]
“Following inquiries, it turns out that the raw data are no longer available having been lost as a result of computer failure.” [85]
“… the original image data for experiments shown in Figs 2, 3, and 7 are no longer available.” [86]
**Problems with Preparing or Analyzing Data**
“Through the automated process of the analysis the authors mistakenly failed to identify that these values were inverse values, and thus, the direction of changes in the individual gene analysis is opposite to those reported in the article.” [87]
“While there are no concerns about the data themselves, the experimental and control groups were inadvertently switched during the original analysis. This error unfortunately lead to the opposite results being reported.” [43]
“…the responses for ‘attitude’ and ‘intention’ measures were switched and may have influenced the findings from the developed regression model and its results” [42]
“…The purpose of the recoding was to change the randomization assignment variable format of “1, 2” to a binary format of “0, 1.” However, the assignment was made incorrectly and resulted in a reversed coding of the study groups.” [44]
“There was a major error in the coding in their dependent variable of marital status” [88]
“identified a mistake in the way the original data were merged” [89]
“…we had miscoded the National Health and Nutrition Examination Survey variable of trying to lose weight by missing a skip pattern that started in the 1999–2000 survey.” [90]
“…the code created to manually anonymize the data was accidentally run twice. During the first run, anonymized subject identifiers were successfully assigned to both biosamples and clinical data. However, after this first run had passed quality control checks, the anonymization code was re-run inadvertently, replacing the first correct set of identifiers with a random and incorrect set.” [91]
“The models did not include random slopes for the term perceived makeup attractiveness, and we have now learned that the Type 1 error rate can be inflated when by-subject random slopes are not included” [45]
“For analysis of repeatedly-assessed time-related data, the authors used comparison of groups at identical time points. This form of cross-sectional comparison at individual time points is not appropriate as it fails to account for patient variability.” [92]
“the statistical analysis to handle risk factors with more than two categories is incorrect” [93]
“The reason for this decision is that the statistical methodology we used did not adequately limit the impact of outlier data points on our findings. This was evident after reanalysis of the data using a different method.” [94]
“A methodological error has led to immortality bias within the findings of this article; therefore, the survival intervals for participants used in this survey were unsound.” [46]
“…review has confirmed firstly that within-group changes were highlighted rather than between-group differences as appropriate for a randomized trial…” [95]
“the authors discovered statistical errors which need further validation” [96]

Among the notices that identified problems with generating or acquiring data, reasons ranged from problems with instrumentation or measurement (*“technical error in the measurement”*) [36] to misidentified study subjects (*“one of the cell lines […] had been unintentionally misidentified;” “transgenic mice reported […] were misidentified”)*; *“incorrect cohort identification (ie, we missed many patients […]”*) [37–39]. Problems with incorrect data entry (*“incorrectly entered data for six subjects;” “some data points that should have been entered as a positive result were instead entered as having a negative result”*) [40,41] might have been prevented by more robust data capture and data quality checking procedures. In some instances, notices stated that data were no longer available or lost.

Among the retraction notices that identified problems with preparing or analyzing data, errors ranged from simple to complex. Some retraction notices described misclassification errors that are easy to make but may reverse a finding, such as miscoding a binary variable (*“the responses for “attitude” and “intention” measures were switched;” “the experimental and control groups were inadvertently switched;” “the assignment was made incorrectly and resulted in a reversed coding of the study groups”*) [42–44]. Other errors included inappropriate selection of statistical methods (*“the model did not include random slopes;” “immortality bias within the findings;” “did not adequately limit the impact of outlier data points”*) [45–47]. Although some retraction notices explained the specific statistical error, other descriptions were nonspecific, providing little insight into the root cause (*“the authors discovered statistical errors”*) [48].

### Characteristics of retracted articles

#### Authorship

Among 786 full-text articles that were reviewed, most had multiple authors, but only 302 (38%) included a statement of authors’ contributions. The median (interquartile range) number of individual authors was 6 (4-8) (Table 3). Very few retracted articles included a consortium in the author list (*n* = 11, 1%).

**Table 3.** Characteristics of 786 retracted articles in clinical and translational research

Characteristic	*N*	(%)
**Authorship**		
Consortium listed as an author	11	
Attribution statement	302	
Number of authors, median (IQR)	6	
**Data**		
Primary type of data used		
Previously Collected for Research Purposes	91	
Previously Collected, not Necessarily for Research	87	
*De novo* Collected	608	
Undefined	580	
Entered into Editable File (e.g. Excel, CSV, Tab Delimited)	18	
Database Program (e.g. ACCESS, SQL)	3	
Research Study Software (e.g. REDCap, CTMS)	2	
Other	5	
Data availability		
Publicly available	67	
Available upon request	21	
Unknown	698	
**Study Design** ^1^		
Systematic review or meta-analysis	47	
Use of live animals	213	
Human subjects research	571	
Clinical Trial	80	
Benchtop (e.g. Cell lines, tissue)	213	
Observational Study	316	
Case-Control	29	
Cross Sectional	135	
Longitudinal / Repeated Measures	120	
Case Report	29	
Undefined	3	
**Methods and Analysis**		
Prespecified statistical analysis plan		
Yes, publicly available	5	
Yes, stated as prespecified but not publicly available	7	
Not explicitly stated	774	
Statistical analysis software^1^		
Excel, Spreadsheet, or Google Sheets	12	
GraphPad PRISM	86	
JMP	7	
Matlab	11	
Minitab	3	
Python	2	
R or R Studio	29	
SAS	39	
SPSS or PASW	229	
Stata	43	
Statistica	4	
Undefined	300	
Other^2^	64	
Reproducible research methods	9	

1 Response categories for these questions were not mutually exclusive.

2 e.g., Comprehensive Meta-Analysis, Origin, SPM (Statistical Parametric Mapping), StatView.

#### Data

Although most articles collected primary data, there were few details about methods for data capture or data availability. More than three-quarters of articles reported collecting data *de novo* (*n* = 608, 77%). The remaining articles included data that were either previously collected for research purposes (*n* = 91, 12%), such as publicly available datasets (e.g., NHANES, BRFSS) and meta-analyses, or data that were previously collected but not necessarily for research purposes (*n* = 87, 11%), such as electronic health records and insurance claim data.

Of the 608 articles that collected data *de novo*, most did not report the methods used for data collection and storage (*n* = 580, 95%). A few articles reported using Microsoft Excel spreadsheets or other editable files (e.g., CSV or tab delimited) for data collection (*n* = 18, 3%); less than 1% reported using data collection tools such as REDCap, clinical trial management software, or database programs such as Microsoft Access or SQL.

Most articles (*n* = 698, 89%) did not include any statements about data availability. Fewer than 1 in 10 articles included data that were publicly available (i.e., either the original data source was public or the authors made their *de novo* data available). Less than 3% of articles (*n* = 21) explicitly stated that the data were available upon request.

#### Study design

Approximately three-quarters of articles involved human research (*n* = 571, 77%), about a quarter involved animal research (*n* = 213, 29%), and 47 (6%) were systematic reviews or meta-analyses (Table 3). Within the retracted articles coded as human research, 316 (55%) included an observational study, 213 (37%) described benchtop data, and 80 (14%) involved a clinical trial based on the current National Institutes of Health definition [49].

### Methods and analysis

Details that support replication and reproduction were infrequently reported. More than one-third of the retracted articles did not specify the statistical analysis software used (*n* = 300, 38%). The most common programs were SPSS/PASW (*n* = 229, 29%) and GraphPad PRISM (*n* = 86, 11%). Statistical software such as Stata (*n* = 43, 5%), SAS (*n* = 39, 5%), or R or R Studio (*n* = 29, 4%) were infrequently reported. A total of 12 articles (2%) mentioned a prespecified statistical analysis plan and only five stated it was publicly available (Table 3). Only nine articles (1%) mentioned additional tools to support reproducing analyses and transparency; four of these specifically referenced Open Science Framework, other articles reported sharing code/scripts [50].

## Discussion

To our knowledge, this is the first comprehensive scoping review of articles in clinical and translational research that were retracted for errors in data capture, management, and/or analysis. Among the retracted articles, we observed a pervasive lack of reporting on data capture, management, storage, and statistical software. While some retraction notices provided detailed information about the discovery and provenance of errors, others provided limited or no actionable information for other investigators to learn from.

### Reasons for retracting articles

Our findings highlight the need for greater attention to data acquisition. Nearly half of retraction notices (42%) described issues with generating or acquiring data. Similar to the 87% of retracted articles reported in MEDLINE retraction notices [2], the majority of retracted articles (77%) involved *de novo* data collection. However, only a small fraction of these articles (5%) described how *de novo* data were captured and stored. Among those few articles that did, more named all-purpose business spreadsheet tools, such as Microsoft Excel, than programs specifically designed for robust research data capture, such as REDCap or clinical trial management systems. Research teams should consider the capture and storage of their (hard won) research data as a critical, if not especially exciting step, in the research process, and one that will require even more attention as data sets become larger and data elements more complex.

Data sharing was the exception rather than the norm – only one in ten articles made statements about data availability; even fewer stated data were publicly available. We expect these percentages will increase as many entities within the scientific community, including journals and funding agencies, require that data be made available through supplemental files or data repositories. For example, the National Institutes of Health recently issued a policy requiring data management and sharing plans in all grant applications submitted on or after January 25, 2023 [51]. However, in order for shared data to facilitate secondary analyses, it must be accompanied by accurate codebooks and detailed documentation. Data sharing makes it possible to uncover errors in previous analyses, but it also allows errors from data collection to propagate beyond the original investigation. Data with protected health information and personally identifying information – common in clinical and translational research – requires special considerations when sharing [52,53].

Whether or not a research group intends to share its data, collecting and documenting it as if it will be shared will only benefit the work. Data management software, such as REDCap, that includes data validation, built-in data quality checks, and options for double data entry, may help avoid errors in data entry and improve data quality. In addition, many of these programs can automatically generate codebooks and well-documented datasets. The documentation may reduce data preparation errors such as miscoding a binary variable (e.g., reversing negative/positive, present/absent).

Software is an integral part of the research process, yet over one-third (38%) of the retracted articles did not specify the statistical analysis software used, and only a few articles (1%) reported sharing code or using specific tools that support reproducing analyses [54]. Our findings are consistent with a recent study that found open source software such as Python are R have been cited far less frequently in retracted articles than software such as SPSS and GraphPad PRISM [55].

At the time of this writing, a vast array of software tools are available that support reproducible research [56]. For example, tools such as R Markdown, Jupyter Notebooks, SAS ODS, and StatTag can all be used to connect manuscript text to analytic results and output, and therefore avoid situations in which an article reports results that are not supported by statistical output [32,56–59]. Tools such as Open Science Framework, mentioned in four retracted articles, provide online and open project documentation and management [50]; GitHub and Code Ocean are online platforms for sharing and running analytic code [60,61]. Although it is not standard practice to cite software tools that support reproducibility and transparency, our results suggest they should be used ubiquitously and regularly. Citing their use may also encourage others to adopt similar tools.

We also recommend that investigators consider using reproducibility checklists or developing their own, both to guard against errors leading to retraction as well as to streamline their research workflow. Checklists vary across disciplines, but typically include reporting prompts to ensure sufficient methodological detail is provided (e.g., are the methods for imputation described?) [62]. Other checklists describe concrete, actionable items related to data capture, file organization, data documentation, computing environments, software used, and data analysis [63,64]. It may help investigators to take a “pragmatic” approach to reproducibility and broadly consider how to account for and document variation and change across the research project [65,66]. For example, documenting how data files are stored and versioned protects against lost data and using the wrong file. Data preparation workflows and analysis methods are increasingly complex – it is not surprising that errors seep into this process. The critical step for research teams is to both prevent and discover errors prior to publication. Checklists may both reduce errors as well as help identify any that persist.

To avoid errors related to inappropriate statistical methods, we recommend that research teams include statisticians or similarly trained individuals with specific expertise and experience in design and conduct of data analysis. Once the team includes appropriate expertise, well-documented analytic code is essential to verify results.

### The retraction process

We found that authors played a substantial role in the retraction process, either by initiating (40%) or formally retracting (58%) the article, which aligns with similar studies of MEDLINE retractions [2,20]. We observed substantially fewer retraction notices that mentioned external investigations compared with prior studies [67]. However, the difference may be owing to our exclusion of articles explicitly identified as scientific misconduct. About one-third of the retraction notices (31%) that we reviewed did not indicate who initiated the retraction, markedly lower than the 55% observed in library and information sciences for published errata [68]. We also note that author involvement in the retraction process may take many forms; we report only on the description in the notice itself, which may not tell the full story. For example, authors may be required by institutional policy to request a retraction, or they may merely have contacted the journal with notice of a correction that then turned into a retraction.

The COPE and the International Committee of Medical Journal Editors have published practice guidelines for retracting articles; both recommend retraction notices including reasons for retraction [31,69]. The COPE guidelines, originally published in 2009 and updated in 2019, are widely endorsed by publishing bodies in order to create uniformity in the retraction process [70]. Despite these recommendations and endorsements, we found wide variation in the content of retraction notices and inconsistent adherence to guidelines.

Retractions with ambiguous language were not only difficult to classify but also led us to speculate that misconduct might have occurred. For example, authors could report that supporting data were “lost” or “no longer available” to cover-up a fabricated figure. “Problems” with data collection or “unreliable data” could be euphemisms for falsification of data. Similarly, authors could report “serious statistical errors” to cover-up data or models that were manipulated to produce a desired p-value. In the past decade, there have been calls to update retraction policies so that retraction notices resulting from honest mistakes may be distinguished from instances of research misconduct [71–73].

In addition to the findings supported by the data we extracted from full-text articles and retraction notices, we also gathered anecdotal information about the visibility of retracted articles through the scoping review process. The PubMed database clearly indicates that an article has been retracted with a red and pink banner and a link to the retraction notice. Some journals include additional, obvious, and helpful visual indicators, such as watermarking retracted articles with “RETRACTED” in large red letters across every page. In contrast, other journals included a small retraction box at the end of the article or failed to indicate the retraction altogether. Some articles were formally retracted and subsequently replaced with a corrected version that confusingly used the same DOI as the original article. This approach made it difficult to locate the retracted version and determine whether an article had been retracted. Even when the formal retraction process follows COPE guidelines and the article is clearly shown as being retracted, it is impossible to inform every person who may have included the retracted article in their citation library. Unfortunately, many articles continue to be mistakenly cited long after they are retracted, sometimes up to decades later [1,74]. The scientific community would benefit from journals ensuring that they adopt practices that clearly indicate if articles are retracted, including issuing a new DOI for a replacement article, and as possible, automating processes to check references and ensure that retracted articles are not being cited.

The absence of transparency in retraction notices limits our ability to learn from them. Although some journals provided a robust addendum of the retraction, breaking down the rationale in great detail, others provided only vague descriptions (*“wrong content with serious consequences;” “serious scientific errors”*) [34,35]. Detailed analysis and reporting of errors align with root cause analysis – an important component of process improvement frameworks that may aid efforts to improve the research process and reduce the volume of future retractions [75,76].

Beyond the purposes of this scoping review, there is value in having retracted articles available online, with the caveat that they are clearly indicated as being retracted. In the context of retract and replace scenarios, The JAMA Network has a method by which the changes are highlighted in the retracted version [77]. This approach makes it very clear to readers where errors occurred, and which content is inaccurate or unreliable; we found their explanations highly informative for qualitative review especially.

We did not specifically capture information on instances of replacement, nor is this information transparent in many cases. Although some retracted articles may eventually be republished with the errors corrected, not all articles are salvageable. We speculate that in general, errors in data preparation or analysis may be corrected more readily than issues in data acquisition, so long as the original data still exist. For example, errors that involve contamination, measurement, or loss of data may not be easily remedied (e.g., *“…we discovered a technical error in the measurement…,” “…underlying data …are unavailable,”* Table 2), suggesting that associated articles cannot be corrected without collecting new data. Less pervasive errors in data collection may be fixed (e.g., *“incorrectly entered data for six subjects on two variables,” “some data points that should have been entered as a positive result were instead entered as having a negative result,”* Table 2) and the article resubmitted. Similarly, errors that involve data preparation or incorrect statistical analyses (e.g., *“identified a mistake in the way the original data were merged,” “the models did not include random slopes,”* Table 2) can be corrected by appropriately analyzing the original data, if available. In instances where data can be salvaged and/or analyses fixed after retraction, we note that articles may need substantial revision prior to submission if findings change in meaningful ways.

### Limitations

Our findings generalize to retracted articles, which constitute a biased subset of articles with errors. For example, retracted articles may be more likely to contain noticeable errors, especially errors in figures, than articles that have not been retracted. Post hoc analyses indicated there were 246 retraction notices (28%) that referenced “image” or “figure.” We found that passive voice and euphemistic language are common in retraction notices – wording reflects a political and legal process that limits transparent documentation. Due to the ambiguity of retraction notices, we could not reliably classify reasons for retraction beyond our two categories. The purpose of our scoping review was descriptive and did not include a control group of articles that had not been retracted. Because the typical time to retraction is longer than 1 year and we included articles published up to the date of our data pull, our results may not be representative of more recent retractions across clinical and translational research, especially for articles published during the COVID-19 pandemic. In fact, there was a dip in time to retraction during the COVID-19 pandemic that we did not capture in our findings because our data pull preceded the pandemic [78].

Our inclusion/exclusion criteria for abstract review relied on the coded subject lists in the RWDB; it was infeasible to review the titles or abstracts themselves to determine if the coded subjects formed a substantive component of the reported research. Our inclusion/exclusion criteria for abstract review also relied on the initial categorizations of retraction reasons in the RWDB – definitions that are subjective and may shift over time. Although we obtained 89% of the articles eligible for full-text review (786 out of 884), our sample may have been biased based on the availability of articles within our institution and in the public domain. We did not assess how many articles that were retracted were eventually republished. Despite these limitations, our findings have implications for the scientific community.

## Conclusions

The scoping review identified more than 800 articles in clinical and translational research retracted over 10 years for concerns related to data capture, management, and/or analysis. Authors have the opportunity to improve the rigor of scientific research by reporting methods for data capture and management, statistical software, and other software tools or code sharing that support transparency and reproducibility. Journals, editors, and peer reviewers can contribute to these improvements by advocating for widespread adoption of this documentation. In addition, journals have the opportunity to help the scientific record self-correct by requiring detailed, transparent retraction notices.

## Supporting information

Baldridge et al. supplementary material Baldridge et al. supplementary material

## Supplementary material

For supplementary material accompanying this paper visit https://doi.org/10.1017/S2059866124005338. click here to view supplementary material
