# Supplementary material for: The epidemiology of errors in data capture, management, and analysis: A scoping review of retracted articles and retraction notices in clinical and translational research
Source: J Clin Transl Sci. 2024 May 17;8(1):e110. doi: 10.1017/cts.2024.533 (PMC11626570; doi:10.1017/cts.2024.533)
Supplement: Baldridge et al. supplementary material [file S2059866124005338sup001.pdf]

Online Supplement

The Epidemiology of Errors in Data Capture, Management, and Analysis: A Scoping Review of Retracted Articles and Retraction Notices in Clinical and Translational Research

Abigail S. Baldridge, Grace C. Bellinger, Oriana M. Fleming, Luke V. Rasmussen, Eric W. Whitley, and Leah J. Welty

Table 1. List of Unique Subjects and Database Frequencies.

Subject	Frequency	Subject to Include	Subject to Exclude
(B/T) Business - Accounting	250		
(B/T) Business - Economics	1,975		
(B/T) Business - General	877		
(B/T) Business - Management	1,696		
(B/T) Business - Manufacturing	76		
(B/T) Business - Marketing	335		
(B/T) Business - Public Relations	82		
(B/T) Computer Science	1,875		
(B/T) Foreign Aid	5		Yes
(B/T) Government	616		Yes
(B/T) International Relations	188		Yes
(B/T) Technology	2,681		
(B/T) Transportation	389		
(B/T) Urban Planning	261		
(BLS) Agriculture	335		Yes
(BLS) Anatomy/Physiology	532	Yes	
(BLS) Anthropology	27	Yes	
(BLS) Archeology	24		Yes
(BLS) Biochemistry	2,280	Yes	
(BLS) Biology - Cancer	1,315	Yes	
(BLS) Biology - Cellular	3,487	Yes	
(BLS) Biology - General	636	Yes	
(BLS) Biology - Molecular	2,290	Yes	
(BLS) Forensic Sciences	6		Yes
(BLS) Genetics	1,893	Yes	
(BLS) Microbiology	698	Yes	
(BLS) Neuroscience	551	Yes	
(BLS) Nutrition	140	Yes	
(BLS) Parasitology	63	Yes	
(BLS) Plant Biology/Botany	550		
(BLS) Toxicology	492	Yes	
(BLS) Zoology	381		
(ENV) Climate Change	48		
(ENV) Climatology	69		
(ENV) Ecology	415		
(ENV) Environmental Sciences	792		
(ENV) Food Science	107		
(ENV) Ground/Surface Water	299		
(HSC) Biostatistics/Epidemiology	254	Yes	
(HSC) Medicine - Alternative	115	Yes	
(HSC) Medicine - Anesthesia/Anaesthesia	365	Yes	
(HSC) Medicine - Cardiology	412	Yes	

(HSC) Medicine - Cardiovascular	605	Yes	
(HSC) Medicine - Dentistry	170	Yes	
(HSC) Medicine - Dermatology	114	Yes	
(HSC) Medicine - Diabetes	215	Yes	
(HSC) Medicine - Drug Design	358	Yes	
(HSC) Medicine - Endocrinology	229	Yes	
(HSC) Medicine - Gastroenterology	434	Yes	
(HSC) Medicine - General	229	Yes	
(HSC) Medicine - Geriatric	103	Yes	
(HSC) Medicine - Immunology	593	Yes	
(HSC) Medicine - Infectious Disease	329	Yes	
(HSC) Medicine - Internal	62	Yes	
(HSC) Medicine - Neurology	550	Yes	
(HSC) Medicine - Nursing	50	Yes	
(HSC) Medicine - Obstetrics/Gynecology	475	Yes	
(HSC) Medicine - Oncology	1,063	Yes	
(HSC) Medicine - Ophthalmology	125	Yes	
(HSC) Medicine - Orthopedics	368	Yes	
(HSC) Medicine - Otorhinolaryngology	146	Yes	
(HSC) Medicine - Pathology	411	Yes	
(HSC) Medicine - Pediatrics	268	Yes	
(HSC) Medicine - Pharm	1	Yes	
(HSC) Medicine - Pharmacology	788	Yes	
(HSC) Medicine - Psychiatry	140	Yes	
(HSC) Medicine - Pulmonology	254	Yes	
(HSC) Medicine - Rehabilitation/Therapy	476	Yes	
(HSC) Medicine - Sports	73	Yes	
(HSC) Medicine - Surgery	790	Yes	
(HSC) Medicine - Transplantation	33	Yes	
(HSC) Medicine - Urology/Nephrology	260	Yes	
(HSC) Nutrition	58	Yes	
(HSC) Occupational Health and Safety	93	Yes	
(HSC) Public Health and Safety	423	Yes	
(HSC) Radiology/Imaging	257	Yes	
(HSC) Sports Science	17	Yes	
(HSC) Veterinary Science	118		Yes
(HUM) Architecture	92		Yes
(HUM) Arts - Biography	1		Yes
(HUM) Arts - Film Studies	11		Yes
(HUM) Arts - General	72		Yes
(HUM) Arts - Music	8		
(HUM) Arts -Literature/Poetry	70		
(HUM) Cartography	14		Yes
(HUM) History - Africa	2		Yes

(HUM) History - Asia	25		Yes
(HUM) History - Europe	26		Yes
(HUM) History - General	21		Yes
(HUM) History - North America	5		Yes
(HUM) History - South America	4		Yes
(HUM) History - United States	2		Yes
(HUM) Journalism	6		Yes
(HUM) Philosophy	41		Yes
(HUM) Religion	69		Yes
(PHY) Astronomy	13		Yes
(PHY) Astrophysics	52		Yes
(PHY) Chemistry	1,176		
(PHY) Cosmology	15		Yes
(PHY) Crystallography/Spectroscopy	318		Yes
(PHY) Energy	279		Yes
(PHY) Engineering - Chemical	424		Yes
(PHY) Engineering - Electrical	713		Yes
(PHY) Engineering - General	501		Yes
(PHY) Engineering - Mechanical	619		Yes
(PHY) Engineering - Structural	313		Yes
(PHY) Forensic Sciences	3		Yes
(PHY) Geology	306		Yes
(PHY) Hydrology	110		Yes
(PHY) Materials Science	1,042		Yes
(PHY) Mathematics	3,074		Yes
(PHY) Nanotechnology	229		
(PHY) Physics	663		
(PHY) Statistics	128		
(PUB) Institutional Journals	100		
(PUB) Society/Association Journals	4,094		
(SOC) Communications	205		
(SOC) Criminology	36		
(SOC) Education	1,612		
(SOC) Ethics/Bioethics	71		Yes
(SOC) Forensics	9		Yes
(SOC) Law/Legal Issues	144		Yes
(SOC) Linguistics	65		
(SOC) Military/Naval Studies	56		Yes
(SOC) Philosophy	20		Yes
(SOC) Political Science	49		Yes
(SOC) Psychology	574	Yes	
(SOC) Sexual And Marital Therapy	21		
(SOC) Sociology	441	Yes	
(SOC) Sports and Recreation	48		

Table 2. List of Reasons for Retraction of 12,555 Publications in BLS, HSC, or SOC.

Reason	Frequency	Reason to Include	Reason to Exclude
Author Unresponsive;	124		
Breach of Policy by Author;	275		
Breach of Policy by Third Party;	3		
Cites Prior Retracted Work;	60		
Civil Proceedings;	19		
Complaints about Author;	30		
Complaints about Company/Institution;	2		
Complaints about Third Party;	6		
Concerns/Issues About Authorship;	262		
Concerns/Issues About Data;	778	Yes	
Concerns/Issues About Image;	178		
Concerns/Issues about Referencing/Attributions;	164		
Concerns/Issues About Results;	263	Yes	
Concerns/Issues about Third Party Involvement;	38		
Conflict of Interest;	76		
Contamination of Cell Lines/Tissues;	48		
Contamination of Materials (General);	26		
Contamination of Reagents;	9		
Copyright Claims;	162		
Criminal Proceedings;	48		Yes
Date of Retraction/Other Unknown;	299		
Doing the Right Thing;	51	Yes	
Duplication of Article;	1,041		
Duplication of Data;	250		
Duplication of Image;	804		
Duplication of Text;	206		
Error by Journal/Publisher;	423		
Error by Third Party;	26		
Error in Analyses;	416	Yes	
Error in Cell Lines/Tissues;	45		
Error in Data;	787	Yes	
Error in Image;	347		
Error in Image;Notice - No/Limited Information;	1		
Error in Materials (General);	83		
Error in Methods;	448		
Error in Results and/or Conclusions;	454	Yes	
Error in Text;	93	Yes	
Ethical Violations by Author;	305		Yes
Ethical Violations by Third Party;	23		Yes
Euphemisms for Duplication;	242		Yes
Euphemisms for Misconduct;	19		Yes

Euphemisms for Misconduct;Notice - No/Limited Info	1	Yes
Euphemisms for Plagiarism;	438	Yes
Fake Peer Review;	419	Yes
Falsification/Fabrication of Data;	892	Yes
Falsification/Fabrication of Image;	324	
Falsification/Fabrication of Results;	80	
Forged Authorship;	204	Yes
Hoax Paper;	6	Yes
Informed/Patient Consent - None/Withdrawn;	90	
Investigation by Company/Institution;	1,548	
Investigation by Journal/Publisher;	1,162	
Investigation by ORI;	211	
Investigation by Third Party;	345	
Lack of Approval from Author;	181	
Lack of Approval from Company/Institution;	88	
Lack of Approval from Third Party;	98	
Lack Of Balance/Bias Issues;	30	
Lack of IRB/IACUC Approval;	247	
Legal Reasons/Legal Threats;	45	Yes
Manipulation of Images;	525	
Manipulation of Results;	27	
Miscommunication by Author;	55	
Miscommunication by Author;Notice - No/Limited Inf	1	
Miscommunication by Company/Institution;	5	
Miscommunication by Journal/Publisher;	10	
Miscommunication by Third Party;	8	
Misconduct - Official Investigation/Finding;	861	Yes
Misconduct by Author;	1,034	Yes
Misconduct by Company/Institution;	5	Yes
Misconduct by Third Party;	20	Yes
No Further Action;	2	
Nonpayment of Fees/Refusal to Pay;	7	
Not Presented at Conference;	6	
Notice - Lack of;	156	
Notice - Limited or No Information;	1,245	
Notice - No/Limited Information;	29	
Notice - Unable to Access via current resources;	129	
Objections by Author(s);	210	
Objections by Company/Institution;	20	
Objections by Third Party;	190	
Plagiarism of Article;	617	Yes
Plagiarism of Data;	132	Yes
Plagiarism of Image;	111	Yes
Plagiarism of Text;	437	Yes

Publishing Ban;	31		
Results Not Reproducible;	365	Yes	
Retract and Replace;	137		
Sabotage of Materials;	1		Yes
Salami Slicing;	10		
Taken from Dissertation/Thesis;	5		
Taken via Peer Review;	3		
Temporary Removal;	58		
Unreliable Data;	384		
Unreliable Image;	120		
Unreliable Results;	762	Yes	
Updated to Retraction;	2		
Upgrade/Update of Prior Notice;	165		
Withdrawal;	681		
Withdrawal;Notice - No/Limited Information;	2		
Withdrawn to Publish in Different Journal;	126		

Table 3. Cohen's Kappa for Inter-Reviewer Agreement of Full Text Review Questions in a Random Sample (n=44).

Data Element	Cohen's Kappa
Consortia	NaN*
Author Number	0.97
Full Text Available	0.81
Author Attribution	0.90
Data Type	0.84
Data Storage	0.87
Data Availability	0.78
Systematic Review	0.86
Human Subjects Design	0.89
Study Design	0.86
Observational	0.73
Temporality	0.78
Animal Study	0.93
Statistical Analysis Plan	0.83
Software State	0.81
Reproducible Methods	0.80

*Kappa calculation is NaN since all responses matched and were the same ("No") for every question in the sample.

Preferred Reporting Items for Systematic reviews and Meta-Analyses extension for Scoping Reviews (PRISMA-ScR) Checklist

SECTION	ITEM	PRISMA-ScR CHECKLIST ITEM	REPORTED ON PAGE #
**TITLE**			
Title	1	Identify the report as a scoping review.	1
**ABSTRACT**			
Structured summary	2	Provide a structured summary that includes (as applicable): background, objectives, eligibility criteria, sources of evidence, charting methods, results, and conclusions that relate to the review questions and objectives.	3
**INTRODUCTION**			
Rationale	3	Describe the rationale for the review in the context of what is already known. Explain why the review questions/objectives lend themselves to a scoping review approach.	4-6
Objectives	4	Provide an explicit statement of the questions and objectives being addressed with reference to their key elements (e.g., population or participants, concepts, and context) or other relevant key elements used to conceptualize the review questions and/or objectives.	5-6
**METHODS**			
Protocol and registration	5	Indicate whether a review protocol exists; state if and where it can be accessed (e.g., a Web address); and if available, provide registration information, including the registration number.	6
Eligibility criteria	6	Specify characteristics of the sources of evidence used as eligibility criteria (e.g., years considered, language, and publication status), and provide a rationale.	7-9
Information sources*	7	Describe all information sources in the search (e.g., databases with dates of coverage and contact with authors to identify additional sources), as well as the date the most recent search was executed.	6,7,9-11
Search	8	Present the full electronic search strategy for at least 1 database, including any limits used, such that it could be repeated.	6,7,9-11
Selection of sources of evidence†	9	State the process for selecting sources of evidence (i.e., screening and eligibility) included in the scoping review.	6,7,9-11
Data charting process‡	10	Describe the methods of charting data from the included sources of evidence (e.g., calibrated forms or forms that have been tested by the team before their use, and whether data charting was done independently or in duplicate) and any processes for obtaining and confirming data from investigators.	9-11
Data items	11	List and define all variables for which data were sought and any assumptions and simplifications made.	9-11
Critical appraisal of individual sources of evidence§	12	If done, provide a rationale for conducting a critical appraisal of included sources of evidence; describe the methods used and how this information was used in any data synthesis (if appropriate).	NA

St. Michael's
Inspired Care.
Inspiring Science.

SECTION	ITEM	PRISMA-ScR CHECKLIST ITEM	REPORTED ON PAGE #
Synthesis of results	13	Describe the methods of handling and summarizing the data that were charted.	12
**RESULTS**			
Selection of sources of evidence	14	Give numbers of sources of evidence screened, assessed for eligibility, and included in the review, with reasons for exclusions at each stage, ideally using a flow diagram.	12, Figure 1
Characteristics of sources of evidence	15	For each source of evidence, present characteristics for which data were charted and provide the citations.	12-17
Critical appraisal within sources of evidence	16	If done, present data on critical appraisal of included sources of evidence (see item 12).	NA
Results of individual sources of evidence	17	For each included source of evidence, present the relevant data that were charted that relate to the review questions and objectives.	12-17
Synthesis of results	18	Summarize and/or present the charting results as they relate to the review questions and objectives.	12-17
**DISCUSSION**			
Summary of evidence	19	Summarize the main results (including an overview of concepts, themes, and types of evidence available), link to the review questions and objectives, and consider the relevance to key groups.	17-22
Limitations	20	Discuss the limitations of the scoping review process.	22-23
Conclusions	21	Provide a general interpretation of the results with respect to the review questions and objectives, as well as potential implications and/or next steps.	23
**FUNDING**			
Funding	22	Describe sources of funding for the included sources of evidence, as well as sources of funding for the scoping review. Describe the role of the funders of the scoping review.	23

JBI = Joanna Briggs Institute; PRISMA-ScR = Preferred Reporting Items for Systematic reviews and Meta-Analyses extension for Scoping Reviews.

* Where *sources of evidence* (see second footnote) are compiled from, such as bibliographic databases, social media platforms, and Web sites.

† A more inclusive/heterogeneous term used to account for the different types of evidence or data sources (e.g., quantitative and/or qualitative research, expert opinion, and policy documents) that may be eligible in a scoping review as opposed to only studies. This is not to be confused with *information sources* (see first footnote).

‡ The frameworks by Arksey and O'Malley (6) and Levac and colleagues (7) and the JBI guidance (4, 5) refer to the process of data extraction in a scoping review as data charting.

§ The process of systematically examining research evidence to assess its validity, results, and relevance before using it to inform a decision. This term is used for items 12 and 19 instead of "risk of bias" (which is more applicable to systematic reviews of interventions) to include and acknowledge the various sources of evidence that may be used in a scoping review (e.g., quantitative and/or qualitative research, expert opinion, and policy document).

From: Tricco AC, Lillie E, Zarin W, O'Brien KK, Colquhoun H, Levac D, et al. PRISMA Extension for Scoping Reviews (PRISMAScR): Checklist and Explanation. Ann Intern Med. 2018;169:467–473. doi: 10.7326/M18-0850.

Data Dictionary Codebook

06/22/2023 10:48am

	#	Variable / Field Name	Field Label *Field Note*	Field Attributes (Field Type, Validation, Choices, Calculations, etc.)
**Instrument: Article Information (article_information)**				
	1	[record_id]	Record ID	text
	2	[title]	Section Header: *Article Information*<br>Title	notes, Required, Identifier
	3	[institution]	Institution	text
	4	[journal]	Journal	text
	5	[publisher]	Publisher	text
	6	[country]	Country	text
	7	[author]	Authors	notes
	8	[article_type]	Article Type	text
	9	[published]	Section Header: *Publication Information*<br>Date Published	text (date_mdy), Identifier
	10	[publication_doi]	Publication DOI	text
	11	[publication_id]	Publication PubMed ID	text
	12	[retracted]	Section Header: *Retraction Information*<br>Date Retracted	text (date_mdy), Identifier
	13	[url]	Retraction URL	notes
	14	[retraction_doi]	Retraction DOI	text
	15	[retraction_id]	Retraction PubMed ID	text
	16	[article_information_complete]	Section Header: *Form Status*<br>Complete?	dropdown<br>0 Incomplete<br>1 Unverified<br>2 Complete
**Instrument: Article Subjects (article_subjects)**				
	17	[subject_list]	Section Header: *Study Information: Title: [title] Journal: [journal] Country: [country] Authors: [author] Publication Date: [published] Retraction Date: [retracted] Retraction URL: [url] Publication DOI: [publication_doi] Retraction DOI: [retraction_doi]*<br>List of Subjects	notes
	18	[num_subjects]	Number of Subjects	text (integer, Min: 0)
	19	[bt_business_accounting]	(B/T) Business - Accounting	yesno<br>1 Yes<br>0 No
	20	[bt_business_economics]	(B/T) Business - Economics	yesno<br>1 Yes<br>0 No
	21	[bt_business_general]	(B/T) Business - General	yesno<br>1 Yes<br>0 No
	22	[bt_business_management]	(B/T) Business - Management	yesno<br>1 Yes<br>0 No

	23	[bt_business_marketing]	(B/T) Business - Marketing	yesno<br>1 Yes<br>0 No
	24	[bt_computer_science]	(B/T) Computer Science	yesno<br>1 Yes<br>0 No
	25	[bt_technology]	(B/T) Technology	yesno<br>1 Yes<br>0 No
	26	[bt_transportation]	(B/T) Transportation	yesno<br>1 Yes<br>0 No
	27	[bt_urban_planning]	(B/T) Urban Planning	yesno<br>1 Yes<br>0 No
	28	[bls_anatomyphysiology]	(BLS) Anatomy/Physiology	yesno<br>1 Yes<br>0 No
	29	[bls_anthropology]	(BLS) Anthropology	yesno<br>1 Yes<br>0 No
	30	[bls_biochemistry]	(BLS) Biochemistry	yesno<br>1 Yes<br>0 No
	31	[bls_biology_cancer]	(BLS) Biology - Cancer	yesno<br>1 Yes<br>0 No
	32	[bls_biology_cellular]	(BLS) Biology - Cellular	yesno<br>1 Yes<br>0 No
	33	[bls_biology_general]	(BLS) Biology - General	yesno<br>1 Yes<br>0 No
	34	[bls_biology_molecular]	(BLS) Biology - Molecular	yesno<br>1 Yes<br>0 No
	35	[bls_genetics]	(BLS) Genetics	yesno<br>1 Yes<br>0 No
	36	[bls_microbiology]	(BLS) Microbiology	yesno<br>1 Yes<br>0 No
	37	[bls_neuroscience]	(BLS) Neuroscience	yesno<br>1 Yes<br>0 No
	38	[bls_nutrition]	(BLS) Nutrition	yesno<br>1 Yes<br>0 No

	39	[bls_parasitology]	(BLS) Parasitology	yesno<br>1 Yes<br>0 No
	40	[bls_plant_biologybotany]	(BLS) Plant Biology/Botany	yesno<br>1 Yes<br>0 No
	41	[bls_toxicology]	(BLS) Toxicology	yesno<br>1 Yes<br>0 No
	42	[bls_zoology]	(BLS) Zoology	yesno<br>1 Yes<br>0 No
	43	[env_climatology]	(ENV) Climatology	yesno<br>1 Yes<br>0 No
	44	[env_environmental_sciences]	(ENV) Environmental Sciences	yesno<br>1 Yes<br>0 No
	45	[env_food_science]	(ENV) Food Science	yesno<br>1 Yes<br>0 No
	46	[hsc_biostatisticsepidemiology]	(HSC) Biostatistics/Epidemiology	yesno<br>1 Yes<br>0 No
	47	[hsc_medicine_alternative]	(HSC) Medicine - Alternative	yesno<br>1 Yes<br>0 No
	48	[hsc_med_anesthesia]	(HSC) Medicine - Anesthesia/Anaesthesia	yesno<br>1 Yes<br>0 No
	49	[hsc_medicine_cardiology]	(HSC) Medicine - Cardiology	yesno<br>1 Yes<br>0 No
	50	[hsc_medicine_cardiovascular]	(HSC) Medicine - Cardiovascular	yesno<br>1 Yes<br>0 No
	51	[hsc_medicine_dentistry]	(HSC) Medicine - Dentistry	yesno<br>1 Yes<br>0 No
	52	[hsc_medicine_dermatology]	(HSC) Medicine - Dermatology	yesno<br>1 Yes<br>0 No
	53	[hsc_medicine_diabetes]	(HSC) Medicine - Diabetes	yesno<br>1 Yes<br>0 No
	54	[hsc_medicine_drug_design]	(HSC) Medicine - Drug Design	yesno<br>1 Yes<br>0 No

	55	[hsc_medicine_endocrinology]	(HSC) Medicine - Endocrinology	yesno<br>1 Yes<br>0 No
	56	[hsc_medicine_gastroenterology]	(HSC) Medicine - Gastroenterology	yesno<br>1 Yes<br>0 No
	57	[hsc_medicine_general]	(HSC) Medicine - General	yesno<br>1 Yes<br>0 No
	58	[hsc_medicine_geriatric]	(HSC) Medicine - Geriatric	yesno<br>1 Yes<br>0 No
	59	[hsc_medicine_immunology]	(HSC) Medicine - Immunology	yesno<br>1 Yes<br>0 No
	60	[hsc_medicine_infectious_disease]	(HSC) Medicine - Infectious Disease	yesno<br>1 Yes<br>0 No
	61	[hsc_medicine_internal]	(HSC) Medicine - Internal	yesno<br>1 Yes<br>0 No
	62	[hsc_medicine_neurology]	(HSC) Medicine - Neurology	yesno<br>1 Yes<br>0 No
	63	[hsc_medicine_nursing]	(HSC) Medicine - Nursing	yesno<br>1 Yes<br>0 No
	64	[hsc_medicine_obgyn]	(HSC) Medicine - Obstetrics/Gynecology	yesno<br>1 Yes<br>0 No
	65	[hsc_medicine_oncology]	(HSC) Medicine - Oncology	yesno<br>1 Yes<br>0 No
	66	[hsc_medicine_ophthalmology]	(HSC) Medicine - Ophthalmology	yesno<br>1 Yes<br>0 No
	67	[hsc_medicine_orthopedics]	(HSC) Medicine - Orthopedics	yesno<br>1 Yes<br>0 No
	68	[hsc_medicine_otorhinolaryngology]	(HSC) Medicine - Otorhinolaryngology	yesno<br>1 Yes<br>0 No
	69	[hsc_medicine_pathology]	(HSC) Medicine - Pathology	yesno<br>1 Yes<br>0 No
	70	[hsc_medicine_pediatrics]	(HSC) Medicine - Pediatrics	yesno<br>1 Yes<br>0 No

	71	[hsc_medicine_pharmacology]	(HSC) Medicine - Pharmacology	yesno<br>1 Yes<br>0 No
	72	[hsc_medicine_psychiatry]	(HSC) Medicine - Psychiatry	yesno<br>1 Yes<br>0 No
	73	[hsc_medicine_pulmonology]	(HSC) Medicine - Pulmonology	yesno<br>1 Yes<br>0 No
	74	[hsc_medicine_rehabtherapy]	(HSC) Medicine - Rehabilitation/Therapy	yesno<br>1 Yes<br>0 No
	75	[hsc_medicine_sports]	(HSC) Medicine - Sports	yesno<br>1 Yes<br>0 No
	76	[hsc_medicine_surgery]	(HSC) Medicine - Surgery	yesno<br>1 Yes<br>0 No
	77	[hsc_nutrition]	(HSC) Nutrition	yesno<br>1 Yes<br>0 No
	78	[hsc_ohs]	(HSC) Occupational Health and Safety	yesno<br>1 Yes<br>0 No
	79	[hsc_public_health_and_safety]	(HSC) Public Health and Safety	yesno<br>1 Yes<br>0 No
	80	[pub_societyassociation_journals]	(PUB) Society/Association Journals	yesno<br>1 Yes<br>0 No
	81	[soc_communications]	(SOC) Communications	yesno<br>1 Yes<br>0 No
	82	[soc_education]	(SOC) Education	yesno<br>1 Yes<br>0 No
	83	[soc_psychology]	(SOC) Psychology	yesno<br>1 Yes<br>0 No
	84	[soc_sexual_and_marital_therapy]	(SOC) Sexual And Marital Therapy	yesno<br>1 Yes<br>0 No
	85	[soc_sociology]	(SOC) Sociology	yesno<br>1 Yes<br>0 No
	86	[env_ecology]	(ENV) Ecology	yesno<br>1 Yes<br>0 No

	87	[env_groundsurface_water]	(ENV) Ground/Surface Water	yesno<br>1 Yes<br>0 No
	88	[hsc_medicine_urologynephrology]	(HSC) Medicine - Urology/Nephrology	yesno<br>1 Yes<br>0 No
	89	[hsc_radiologyimaging]	(HSC) Radiology/Imaging	yesno<br>1 Yes<br>0 No
	90	[phy_chemistry]	(PHY) Chemistry	yesno<br>1 Yes<br>0 No
	91	[phy_physics]	(PHY) Physics	yesno<br>1 Yes<br>0 No
	92	[pub_institutional_journals]	(PUB) Institutional Journals	yesno<br>1 Yes<br>0 No
	93	[soc_criminology]	(SOC) Criminology	yesno<br>1 Yes<br>0 No
	94	[soc_sports_and_recreation]	(SOC) Sports and Recreation	yesno<br>1 Yes<br>0 No
	95	[env_climate_change]	(ENV) Climate Change	yesno<br>1 Yes<br>0 No
	96	[hsc_medicine_transplantation]	(HSC) Medicine - Transplantation	yesno<br>1 Yes<br>0 No
	97	[hsc_sports_science]	(HSC) Sports Science	yesno<br>1 Yes<br>0 No
	98	[phy_nanotechnology]	(PHY) Nanotechnology	yesno<br>1 Yes<br>0 No
	99	[phy_statistics]	(PHY) Statistics	yesno<br>1 Yes<br>0 No
	100	[article_subjects_complete]	Section Header: *Form Status*<br>Complete?	dropdown<br>0 Incomplete<br>1 Unverified<br>2 Complete
**Instrument: Retraction Reasons (retraction_reasons)**				
	101	[reason_list]	Section Header: *Study Information: Title: [title] Journal: [journal] Country: [country] Authors: [author] Publication Date: [published] Retraction Date: [retracted] Retraction URL: [url] Publication DOI: [publication_doi] Retraction DOI: [retraction_doi]*<br>List of Reasons	notes

	102	[num_reasons]	Number of Reasons	text
	103	[author_unresponsive]	Author Unresponsive	yesno<br>1 Yes<br>0 No
	104	[breach_of_policy_by_author]	Breach of Policy by Author	yesno<br>1 Yes<br>0 No
	105	[cites_prior_retracted_work]	Cites Prior Retracted Work	yesno<br>1 Yes<br>0 No
	106	[civil_proceedings]	Civil Proceedings	yesno<br>1 Yes<br>0 No
	107	[complaints_about_author]	Complaints about Author	yesno<br>1 Yes<br>0 No
	108	[complaints_about_co_institution]	Complaints about Company/Institution	yesno<br>1 Yes<br>0 No
	109	[complaints_about_third_party]	Complaints about Third Party	yesno<br>1 Yes<br>0 No
	110	[concerns_issues_about_authorship]	Concerns/Issues About Authorship	yesno<br>1 Yes<br>0 No
	111	[concerns_issues_about_data]	Concerns/Issues About Data	yesno<br>1 Yes<br>0 No
	112	[concerns_issues_about_image]	Concerns/Issues About Image	yesno<br>1 Yes<br>0 No
	113	[concerns_issues_about_results]	Concerns/Issues About Results	yesno<br>1 Yes<br>0 No
	114	[referencing_attributions]	Concerns/Issues about Referencing/Attributions	yesno<br>1 Yes<br>0 No
	115	[conflict_of_interest]	Conflict of Interest	yesno<br>1 Yes<br>0 No
	116	[contamination_of_cell_tissues]	Contamination of Cell Lines/Tissues	yesno<br>1 Yes<br>0 No
	117	[contamination_of_materials_gen]	Contamination of Materials (General)	yesno<br>1 Yes<br>0 No

	118	[copyright_claims]	Copyright Claims	yesno<br>1 Yes<br>0 No
	119	[date_of_retraction_other_unknown]	Date of Retraction/Other Unknown	yesno<br>1 Yes<br>0 No
	120	[doing_the_right_thing]	Doing the Right Thing	yesno<br>1 Yes<br>0 No
	121	[duplication_of_article]	Duplication of Article	yesno<br>1 Yes<br>0 No
	122	[duplication_of_data]	Duplication of Data	yesno<br>1 Yes<br>0 No
	123	[duplication_of_image]	Duplication of Image	yesno<br>1 Yes<br>0 No
	124	[duplication_of_text]	Duplication of Text	yesno<br>1 Yes<br>0 No
	125	[error_by_journal_publisher]	Error by Journal/Publisher	yesno<br>1 Yes<br>0 No
	126	[error_by_third_party]	Error by Third Party	yesno<br>1 Yes<br>0 No
	127	[error_in_analyses]	Error in Analyses	yesno<br>1 Yes<br>0 No
	128	[error_in_cell_lines_tissues]	Error in Cell Lines/Tissues	yesno<br>1 Yes<br>0 No
	129	[error_in_data]	Error in Data	yesno<br>1 Yes<br>0 No
	130	[error_in_image]	Error in Image	yesno<br>1 Yes<br>0 No
	131	[error_in_materials_general]	Error in Materials (General)	yesno<br>1 Yes<br>0 No
	132	[error_in_methods]	Error in Methods	yesno<br>1 Yes<br>0 No
	133	[error_in_results_or_conclusions]	Error in Results and/or Conclusions	yesno<br>1 Yes<br>0 No

	134	[error_in_text]	Error in Text	yesno<br>1 Yes<br>0 No
	135	[falsification_fabrication_image]	Falsification/Fabrication of Image	yesno<br>1 Yes<br>0 No
	136	[investigation_by_co_inst]	Investigation by Company/Institution	yesno<br>1 Yes<br>0 No
	137	[investigation_by_journal]	Investigation by Journal/Publisher	yesno<br>1 Yes<br>0 No
	138	[lack_of_balance_bias_issues]	Lack Of Balance/Bias Issues	yesno<br>1 Yes<br>0 No
	139	[lack_of_approval_from_author]	Lack of Approval from Author	yesno<br>1 Yes<br>0 No
	140	[lack_of_approval_third_party]	Lack of Approval from Third Party	yesno<br>1 Yes<br>0 No
	141	[lack_of_irb_iacuc_approval]	Lack of IRB/IACUC Approval	yesno<br>1 Yes<br>0 No
	142	[manipulation_of_images]	Manipulation of Images	yesno<br>1 Yes<br>0 No
	143	[notice_limited_or_no_information]	Notice - Limited or No Information	yesno<br>1 Yes<br>0 No
	144	[objections_by_authors]	Objections by Author(s)	yesno<br>1 Yes<br>0 No
	145	[objections_by_third_party]	Objections by Third Party	yesno<br>1 Yes<br>0 No
	146	[results_not_reproducible]	Results Not Reproducible	yesno<br>1 Yes<br>0 No
	147	[retract_and_replace]	Retract and Replace	yesno<br>1 Yes<br>0 No
	148	[unreliable_data]	Unreliable Data	yesno<br>1 Yes<br>0 No
	149	[unreliable_results]	Unreliable Results	yesno<br>1 Yes<br>0 No

	150	[third_party_involvement]	Concerns/Issues about Third Party Involvement	yesno<br>1 Yes<br>0 No
	151	[contamination_of_reagents]	Contamination of Reagents	yesno<br>1 Yes<br>0 No
	152	[informed_patient_consent]	Informed/Patient Consent - None/Withdrawn	yesno<br>1 Yes<br>0 No
	153	[investigation_by_third_party]	Investigation by Third Party	yesno<br>1 Yes<br>0 No
	154	[lack_of_approval]	Lack of Approval from Company/Institution	yesno<br>1 Yes<br>0 No
	155	[manipulation_of_results]	Manipulation of Results	yesno<br>1 Yes<br>0 No
	156	[miscommunication_by_author]	Miscommunication by Author	yesno<br>1 Yes<br>0 No
	157	[objections]	Objections by Company/Institution	yesno<br>1 Yes<br>0 No
	158	[unreliable_image]	Unreliable Image	yesno<br>1 Yes<br>0 No
	159	[upgrade_update_of_prior_notice]	Upgrade/Update of Prior Notice	yesno<br>1 Yes<br>0 No
	160	[withdrawal]	Withdrawal	yesno<br>1 Yes<br>0 No
	161	[miscommunication_by_third_party]	Miscommunication by Third Party	yesno<br>1 Yes<br>0 No
	162	[notice_lack_of]	Notice - Lack of	yesno<br>1 Yes<br>0 No
	163	[investigation_by_ori]	Investigation by ORI	yesno<br>1 Yes<br>0 No
	164	[publishing_ban]	Publishing Ban	yesno<br>1 Yes<br>0 No
	165	[temporary_removal]	Temporary Removal	yesno<br>1 Yes<br>0 No

	166	[miscommunication_by_journal]	Miscommunication by Journal/Publisher	yesno<br>1 Yes<br>0 No
	167	[salami_slicing]	Salami Slicing	yesno<br>1 Yes<br>0 No
	168	[retraction_reasons_complete]	Section Header: *Form Status*<br>Complete?	dropdown<br>0 Incomplete<br>1 Unverified<br>2 Complete
Instrument: **Abstract Review 1** (abstract_review_1) Enabled as survey				
	169	[abstract_review_date_v1]	Section Header: *Study Information: Title: [title] Journal: [journal] Country: [country] Authors: [author] Publication Date: [published] Retraction Date: [retracted] Retraction URL: [url] Publication DOI: [publication_doi] Retraction DOI: [retraction_doi]*<br>Date of Abstract Review	text (date_mdy)
	170	[abstract_reviewer_v1]	Reviewer	radio<br>0 Abigail Baldridge<br>1 Eric Whitely<br>2 Leah Welty<br>3 Luke Rasmussen
	171	[english_v1]	Is the article written in the English language?	yesno<br>1 Yes<br>0 No<br>Custom alignment: LH
	172	[human_research_v1]	Is the article about human subjects research?	yesno<br>1 Yes<br>0 No<br>Custom alignment: LH
	173	[translational_v1]<br>Show the field ONLY if:<br>[human_research_v1] = '0'	If the article is not about human subjects research, is it applicable to the human translational research pipeline?	yesno<br>1 Yes<br>0 No<br>Custom alignment: LH
	174	[elligible_v1]<br>Show the field ONLY if:<br>[english_v1] = '1' and ([human_research_v1] = '1' or [translational_v1] = '1')	This Article is Eligible.	descriptive
	175	[inelligible_v1]<br>Show the field ONLY if:<br>[english_v1] = '0' or ([human_research_v1] = '0' and [translational_v1] = '0')	This Article is Not Eligible.	descriptive
	176	[eiligibility_v1]	Is the article eligible for full text review?	yesno<br>1 Yes<br>0 No
	177	[abstract_review_1_complete]	Section Header: *Form Status*<br>Complete?	dropdown<br>0 Incomplete<br>1 Unverified<br>2 Complete

Instrument: Abstract Review 2 (abstract_review_2) Enabled as survey

	178	[abstract_review_date_v2]	Section Header: *Study Information: Title: [title] Journal: [journal] Country: [country] Authors: [author] Publication Date: [published] Retraction Date: [retracted] Retraction URL: [url] Publication DOI: [publication_doi] Retraction DOI: [retraction_doi]*<br>Date of Abstract Review	text (date_mdy)
	179	[abstract_reviewer_v2]	Reviewer	radio<br>0 Abigail Baldridge<br>1 Eric Whitely<br>2 Leah Welty<br>3 Luke Rasmussen
	180	[english_v2]	Is the article written in the English language?	yesno<br>1 Yes<br>0 No<br>Custom alignment: LH
	181	[human_research_v2]	Is the article about human subjects research?	yesno<br>1 Yes<br>0 No<br>Custom alignment: LH
	182	[translational_v2]<br>Show the field ONLY if:<br>[human_research_v2] = '0'	If the article is not about human subjects research, is it applicable to the human translational research pipeline?	yesno<br>1 Yes<br>0 No<br>Custom alignment: LH
	183	[elligible_v2]<br>Show the field ONLY if:<br>[english_v2] = '1' and ([human_research_v2] = '1' or [translational_v2] = '1')	This Article is Eligible.	descriptive
	184	[inelligible_v2]<br>Show the field ONLY if:<br>[english_v2] = '0' or ([human_research_v2] = '0' and [translational_v2] = '0')	This Article is Not Eligible.	descriptive
	185	[eiligibility_v2]	Is the article eligible for full text review?	yesno<br>1 Yes<br>0 No
	186	[abstract_review_2_complete]	Section Header: *Form Status*<br>Complete?	dropdown<br>0 Incomplete<br>1 Unverified<br>2 Complete

Instrument: Abstract Review 3 (abstract_review_3) Enabled as survey

	187	[abstract_review_date_v3]	Section Header: *Study Information: Title: [title] Journal: [journal] Country: [country] Authors: [author] Publication Date: [published] Retraction Date: [retracted] Retraction URL: [url] Publication DOI: [publication_doi] Retraction DOI: [retraction_doi]*<br>Date of Abstract Review	text (date_mdy)
	188	[abstract_reviewer_v3]	Reviewer	radio<br>0 Abigail Baldridge<br>1 Eric Whitely<br>2 Leah Welty<br>3 Luke Rasmussen<br>4 Group

	189	[english_v3]	Is the article written in the English language?	yesno<br>1 Yes<br>0 No<br><br>Custom alignment: LH
	190	[human_research_v3]	Is the article about human subjects research?	yesno<br>1 Yes<br>0 No<br><br>Custom alignment: LH
	191	[translational_v3]<br><br>Show the field ONLY if:<br>[human_research_v3] = '0'	If the article is not about human subjects research, is it applicable to the human translational research pipeline?	yesno<br>1 Yes<br>0 No<br><br>Custom alignment: LH
	192	[elligible_v3]<br><br>Show the field ONLY if:<br>[english_v3] = '1' and ([human_research_v3] = '1' or [translational_v3] = '1')	This Article is Eligible.	descriptive
	193	[inelligible_v3]<br><br>Show the field ONLY if:<br>[english_v3] = '0' or ([human_research_v3] = '0' and [translational_v3] = '0')	This Article is Not Eligible.	descriptive
	194	[eiligibility_v3]	Is the article eligible for full text review?	yesno<br>1 Yes<br>0 No
	195	[abstract_review_3_complete]	Section Header: *Form Status*<br><br>Complete?	dropdown<br>0 Incomplete<br>1 Unverified<br>2 Complete
**Instrument: Full Text Review (full_text_review) Enabled as survey**				
	196	[full_text_review_date]	Section Header: *Study Information: Title: [title] Journal: [journal] Country: [country] Authors: [author] Publication Date: [published] Retraction Date: [retracted] Retraction URL: [url] Publication DOI: [publication_doi] Retraction DOI: [retraction_doi]*<br><br>Date of Full Text Review	text (date_mdy), Required<br>Field Annotation: @TODAY @HIDDEN-SURVEY
	197	[full_text_reviewer]	Reviewer	radio, Required<br>0 Abigail Baldridge<br>1 Eric Whitely<br>2 Leah Welty<br>3 Luke Rasmussen<br>4 Grace Bellinger<br>5 Oriana Fleming
	198	[consortia]	Section Header: *Author Information*<br><br>Is one or more of the authors a consortia?	yesno, Required<br>1 Yes<br>0 No
	199	[author_num]	Count the total Number of Individual Authors	text (integer, Min: 0), Required
	200	[retraction_notice]	Section Header: *Retracted Article*<br><br>Copy and Paste the Verbatim Retraction Notice Here:<br>*From the journal website*	notes, Required
	201	[notes]	Notes about the article:<br>*Any extenuating circumstances about the article information or availability*	notes

	202	[full_text_available]	Is the full text of the retracted article available?	yesno, Required<br>1 Yes<br>0 No<br><br>Stop actions on 0
	203	[stop]<br><br>Show the field ONLY if:<br>[full_text_available] = '0'	The full text review does not need to be completed. You may exit the survey.	descriptive
	204	[file_upload]<br><br>Show the field ONLY if:<br>[full_text_available] = '1'	Please upload a pdf of the retracted article.<br>*Save/Print to PDF if the article is only available in other forms (HTML)*	file, Required
	205	[header]<br><br>Show the field ONLY if:<br>[full_text_available] = '1'	For all remaining questions, use ONLY the article itself, or its supplemental materials. If information are not included in the article or supplemental materials, these fields can be left blank or as "N/A".	descriptive
	206	[attribauthor]<br><br>Show the field ONLY if:<br>[full_text_available] = '1'	Section Header: *Author Roles and Qualifications*<br>Is there an attribution statement?<br>*This is a statement of which authors are responsible for which parts of the study, analysis, and manuscript. This may also be called "Author Contributions" or "Author Responsibilities"*	yesno, Required<br>1 Yes<br>0 No
	207	[attrib_statement]<br><br>Show the field ONLY if:<br>[attribauthor] = '1'	Copy and Paste the Attribution Statement Here	notes, Required
	208	[data_type]<br><br>Show the field ONLY if:<br>[full_text_available] = '1'	Section Header: *Data Collection*<br>What is the primary type of data used?<br>*This information is typically found in either the abstract or the methods section.*	radio, Required<br>0 De Novo Collected<br>1 Previously Collected for Research Purposes<br>2 Previously Collected, not Necessarily for Research<br>3 Undefined<br>4 Other
	209	[other_data]<br><br>Show the field ONLY if:<br>[data_type] = '4'	List the Other Type of Data	text, Required
	210	[data_storage]<br><br>Show the field ONLY if:<br>[data_type] = '0'	Where was the primary data collected for the publication?	checkbox, Required<br>0 data_storage___0 Entered into Editable File (e.g. Excel, CSV, Tab Delimited)<br>1 data_storage___1 Research Study Software (e.g. REDCap, CTMS)<br>2 data_storage___2 Database Program (e.g. ACCESS, SQL)<br>3 data_storage___3 Undefined<br>4 data_storage___4 Other
	211	[other_data_storage]<br><br>Show the field ONLY if:<br>[data_storage(4)] = '1'	List the Other Data Collection Method/Location	text, Required
	212	[data_source]<br><br>Show the field ONLY if:<br>[data_type] = '1' or [data_typ e] = '2'	For previously collected data, how was the data obtained for this publication? Copy and paste all relevant statements, using a carriage return between separate statements.	notes, Required
	213	[data_availability]<br><br>Show the field ONLY if:<br>[full_text_available] = '1'	Section Header: *Data Availability*<br>Is the data used within this publication available? Note: if it came from a publically available dataset, then yes.	dropdown, Required<br>0 Publicly available<br>1 Available upon request<br>2 Unknown

	214	[sys_review]<br><br>Show the field ONLY if:<br>[full_text_available] = '1'	Section Header: *Methods*<br><br>Is this a Systematic Review or Meta Analysis?	yesno, Required<br>1 Yes<br>0 No
	215	[human_subj]<br><br>Show the field ONLY if:<br>[sys_review] = '0'	Is this Human Subjects research?	yesno, Required<br>1 Yes<br>0 No
	216	[study_design]<br><br>Show the field ONLY if:<br>[human_subj] = '1'	What was the human subjects design?	checkbox, Required<br>0 study_design___0 Human: Clinical Subjects Trial<br>1 study_design___1 Human: Observational Study<br>2 study_design___2 Benchtop (e.g. Cell lines, tissue)<br>99 study_design___99 Undefined
	217	[observational]<br><br>Show the field ONLY if:<br>[study_design(1)] = '1'	What type of observational study was performed?	radio, Required<br>0 Case-Control<br>1 Cross Sectional<br>2 Longitudinal / Repeated Measures<br>4 Case Report<br>3 Undefined
	218	[temporality]<br><br>Show the field ONLY if:<br>[study_design(1)] = '1'	What was the temporality of the observational study?	radio, Required<br>0 Retrospective<br>1 Prospective<br>2 Both<br>3 Undefined<br>4 NA (For Case Reports or Cross Sectional Studies)
	219	[animal]<br><br>Show the field ONLY if:<br>[sys_review] = '0'	Were live animals used in this research study?<br>*Animals were studied, sacrificed, or otherwise involved in the study*	yesno, Required<br>1 Yes<br>0 No
	220	[sap]<br><br>Show the field ONLY if:<br>[full_text_available] = '1'	Does the article state that a statistical analysis plan was pre-specified?	radio, Required<br>1 Yes, publicly available<br>2 Yes, stated as pre-specified but not publicly available<br>3 Not explicitly stated

	221	[software]<br><br>Show the field ONLY if:<br>[full_text_available] = '1'	What software was used for analysis?	checkbox, Required<br>8 | software___8 | Excel / Spreadsheet / Google Sheets<br>5 | software___5 | GraphPad PRISM<br>9 | software___9 | JMP<br>10 | software___10 | KaleidaGraph<br>11 | software___11 | Minitab<br>13 | software___13 | MPlus<br>7 | software___7 | Power BI<br>3 | software___3 | Python<br>1 | software___1 | R / R Studio<br>0 | software___0 | SAS<br>4 | software___4 | SPSS / PASW / PASW18<br>2 | software___2 | Stata<br>12 | software___12 | Statistica<br>6 | software___6 | Tableau<br>999 | software___999 | Undefined<br>777 | software___777 | Other
	222	[other_software]<br><br>Show the field ONLY if:<br>[software(777)] = '1'	List the Other Software	text, Required
	223	[reproducible_methods]<br><br>Show the field ONLY if:<br>[full_text_available] = '1'	Is there any additional information about the use of reproducible research methods?	yesno, Required<br>1 | Yes<br>0 | No
	224	[reproducible_statement]<br><br>Show the field ONLY if:<br>[reproducible_methods] = '1'	Copy and paste the verbatim language about reproducible methods here:	notes, Required
	225	[full_text_review_complete]	Section Header: *Form Status*<br><br>Complete?	dropdown<br>0 | Incomplete<br>1 | Unverified<br>2 | Complete
Instrument: **Full Text Review Verification** (full_text_review_verification) **Enabled as survey**				
	226	[verification]	Section Header: *Study Information: Title: [title] Journal: [journal] Country: [country] Authors: [author] Publication Date: [published] Retraction Date: [retracted] Retraction URL: [url] Publication DOI: [publication_doi] Retraction DOI: [retraction_doi]*<br><br>Randomly selected for full text review verification?	yesno<br>1 | Yes<br>0 | No
	227	[full_text_review_date_2]	Date of Full Text Review	text (date_mdy), Required<br>Field Annotation: @TODAY @HIDDEN-SURVEY
	228	[full_text_reviewer_2]	Reviewer	radio, Required<br>0 | Abigail Baldridge<br>1 | Eric Whitely<br>2 | Leah Welty<br>3 | Luke Rasmussen<br>4 | Grace Bellinger<br>5 | Oriana Fleming
	229	[consortia_2]	Section Header: *Author Information*<br><br>Is one or more of the authors a consortia?	yesno, Required<br>1 | Yes<br>0 | No
	230	[author_num_2]	Count the total Number of Individual Authors	text (integer, Min: 0), Required

	231	[retraction_notice_2]	Section Header: *Retracted Article*<br>Copy and Paste the Verbatim Retraction Notice Here:<br>*From the journal website*	notes, Required
	232	[notes_2]	Notes about the article:<br>*Any extenuating circumstances about the article information or availability*	notes
	233	[full_text_available_2]	Is the full text of the retracted article available?	yesno, Required<br>1 Yes<br>0 No<br>Stop actions on 0
	234	[stop_2]<br>Show the field ONLY if:<br>[full_text_available_2] = '0'	The full text review does not need to be completed. You may exit the survey.	descriptive
	235	[file_upload_2]<br>Show the field ONLY if:<br>[full_text_available_2] = '1'	Please upload a pdf of the retracted article.<br>*Save/Print to PDF if the article is only available in other forms (HTML)*	file, Required
	236	[header_2]<br>Show the field ONLY if:<br>[full_text_available_2] = '1'	For all remaining questions, use ONLY the article itself, or its supplemental materials. If information are not included in the article or supplemental materials, these fields can be left blank or as "N/A".	descriptive
	237	[attribauthor_2]<br>Show the field ONLY if:<br>[full_text_available_2] = '1'	Section Header: *Author Roles and Qualifications*<br>Is there an attribution statement?<br>*This is a statement of which authors are responsible for which parts of the study, analysis, and manuscript. This may also be called "Author Contributions" or "Author Responsibilities"*	yesno, Required<br>1 Yes<br>0 No
	238	[attrib_statement_2]<br>Show the field ONLY if:<br>[attribauthor_2] = '1'	Copy and Paste the Attribution Statement Here	notes, Required
	239	[data_type_2]<br>Show the field ONLY if:<br>[full_text_available_2] = '1'	Section Header: *Data Collection*<br>What is the primary type of data used?<br>*This information is typically found in either the abstract or the methods section.*	radio, Required<br>0 De Novo Collected<br>1 Previously Collected for Research Purposes<br>2 Previously Collected, not Necessarily for Research<br>3 Undefined<br>4 Other
	240	[other_data_2]<br>Show the field ONLY if:<br>[data_type_2] = '4'	List the Other Type of Data	text, Required
	241	[data_storage_2]<br>Show the field ONLY if:<br>[data_type_2] = '0'	Where was the primary data collected for the publication?	checkbox, Required<br>0 data_storage_2___0 Entered into Editable File (e.g. Excel, CSV, Tab Delimited)<br>1 data_storage_2___1 Research Study Software (e.g. REDCap, CTMS)<br>2 data_storage_2___2 Database Program (e.g. ACCESS, SQL)<br>3 data_storage_2___3 Undefined<br>4 data_storage_2___4 Other
	242	[other_data_storage_2]<br>Show the field ONLY if:<br>[data_storage_2(4)] = '1'	List the Other Data Collection Method/Location	text, Required
	243	[data_source_2]<br>Show the field ONLY if:<br>[data_type_2] = '1' or [data_type_2] = '2'	For previously collected data, how was the data obtained for this publication? Copy and paste all relevant statements, using a carriage return between separate statements.	notes, Required

	244	[data_availability_2]<br><br>Show the field ONLY if:<br>[full_text_available_2] = '1'	Section Header: *Data Availability*<br><br>Is the data used within this publication available? Note: if it came from a publically available dataset, then yes.	dropdown, Required<br>0 Publicly available<br>1 Available upon request<br>2 Unknown
	245	[sys_review_2]<br><br>Show the field ONLY if:<br>[full_text_available_2] = '1'	Section Header: *Methods*<br><br>Is this a Systematic Review or Meta Analysis?	yesno, Required<br>1 Yes<br>0 No
	246	[human_subj_2]<br><br>Show the field ONLY if:<br>[sys_review_2] = '0'	Is this Human Subjects research?	yesno, Required<br>1 Yes<br>0 No
	247	[study_design_2]<br><br>Show the field ONLY if:<br>[human_subj_2] = '1'	What was the human subjects design?	checkbox, Required<br>0 study_design_2___0 Human: Clinical Subjects Trial<br>1 study_design_2___1 Human: Observational Study<br>2 study_design_2___2 Benchtop (e.g. Cell lines, tissue)<br>99 study_design_2___99 Undefined
	248	[observational_2]<br><br>Show the field ONLY if:<br>[study_design_2(1)] = '1'	What type of observational study was performed?	radio, Required<br>0 Case-Control<br>1 Cross Sectional<br>2 Longitudinal / Repeated Measures<br>4 Case Report<br>3 Undefined
	249	[temporality_2]<br><br>Show the field ONLY if:<br>[study_design_2(1)] = '1'	What was the temporality of the observational study?	radio, Required<br>0 Retrospective<br>1 Prospective<br>2 Both<br>3 Undefined<br>4 NA (For Case Reports or Cross Sectional Studies)
	250	[animal_2]<br><br>Show the field ONLY if:<br>[sys_review_2] = '0'	Were live animals used in this research study?<br>*Animals were studied, sacrificed, or otherwise involved in the study*	yesno, Required<br>1 Yes<br>0 No
	251	[sap_2]<br><br>Show the field ONLY if:<br>[full_text_available_2] = '1'	Does the article state that a statistical analysis plan was pre-specified?	radio, Required<br>1 Yes, publicly available<br>2 Yes, stated as pre-specified but not publicly available<br>3 Not explicitly stated

	252	[software_2]<br><br>Show the field ONLY if:<br>[full_text_available_2] = '1'	What software was used for analysis?	checkbox, Required<br>8 software_2___8 Excel / Spreadsheet / Google Sheets<br>5 software_2___5 GraphPad PRISM<br>9 software_2___9 JMP<br>10 software_2___10 KaleidaGraph<br>11 software_2___11 Minitab<br>13 software_2___13 MPlus<br>7 software_2___7 Power BI<br>3 software_2___3 Python<br>1 software_2___1 R / R Studio<br>0 software_2___0 SAS<br>4 software_2___4 SPSS / PASW / PASW18<br>2 software_2___2 Stata<br>12 software_2___12 Statistica<br>6 software_2___6 Tableau<br>999 software_2___999 Undefined<br>777 software_2___777 Other
	253	[other_software_2]<br><br>Show the field ONLY if:<br>[software_2(777)] = '1'	List the Other Software	text, Required
	254	[reproducible_methods_2]<br><br>Show the field ONLY if:<br>[full_text_available_2] = '1'	Is there any additional information about the use of reproducible research methods?	yesno, Required<br>1 Yes<br>0 No
	255	[reproducible_statement_2]<br><br>Show the field ONLY if:<br>[reproducible_methods_2] = '1'	Copy and paste the verbatim language about reproducible methods here:	notes, Required
	256	[full_text_review_verification_complete]	Section Header: *Form Status*<br>Complete?	dropdown<br>0 Incomplete<br>1 Unverified<br>2 Complete

Instrument: Qualitative Review (qualitative_review)

	257	[reasons]	Section Header: *Study Information: Title: [title] Journal: [journal] Country: [country] Authors: [author] Publication Date: [published] Retraction Date: [retracted] Retraction URL: [url] Publication DOI: [publication_doi] Retraction DOI: [retraction_doi]*<br>Reason List from Retraction Watch: [reason_list]	descriptive
	258	[retractionnotice]	[retraction_notice]	descriptive
	259	[qual_reviewer]	Reviewer	radio, Required<br>4 Grace Bellinger<br>5 Oriana Fleming<br>0 Abigail Baldridge<br>1 Eric Whitely<br>2 Leah Welty<br>3 Luke Rasmussen
	260	[adequate]	Does the retraction contain adequate information to perform a qualitative review?	yesno<br>1 Yes<br>0 No

	261	[retractor]<br><br>Show the field ONLY if:<br>[adequate] = '1'	Who is specifically named in the retraction statement as retracting the article?	checkbox, Required<br>1 retractor___1 Author(s)<br>2 retractor___2 Ambiguous Editor(s)/Journal/Publisher<br>3 retractor___3 Editor(s)<br>4 retractor___4 Journal<br>5 retractor___5 Publisher<br>6 retractor___6 Other<br>7 retractor___7 Not Stated
	262	[other_retractor]<br><br>Show the field ONLY if:<br>[retractor(6)] = '1'	If Other:	text, Required
	263	[initiator]<br><br>Show the field ONLY if:<br>[adequate] = '1'	Who initiated the investigation or discovered that the retraction should occur?	checkbox, Required<br>1 initiator___1 Author<br>2 initiator___2 Editor(s)/Journal/Publisher<br>3 initiator___3 Letter to Editor<br>4 initiator___4 External Investigation<br>5 initiator___5 Readers<br>6 initiator___6 Unnamed Entity<br>7 initiator___7 Other<br>8 initiator___8 Not Stated
	264	[other_initiator]<br><br>Show the field ONLY if:<br>[initiator(7)] = '1'	If Other:	text, Required
	265	[author_responded]<br><br>Show the field ONLY if:<br>[adequate] = '1'	Were any of the authors involved in the retraction process?	radio<br>1 Yes<br>2 No, the authors were unresponsive<br>3 No / Not stated
	266	[agree]<br><br>Show the field ONLY if:<br>[adequate] = '1' and [author_responded] = '1'	Do any of the authors disagree with the retraction?	radio, Required<br>1 Yes<br>2 No<br>3 Not stated
	267	[errors]<br><br>Show the field ONLY if:<br>[adequate] = '1'	What types of errors are described in the retraction notice?	checkbox, Required<br>1 errors___1 Data Collection, Capture, Unreliable, or Unverifiable Data<br>2 errors___2 Data Cleaning, Preparation, or Variable Coding<br>3 errors___3 Data No Longer Available or Lost<br>4 errors___4 Error in Study Conduct, Protocol Violation, Ethics<br>5 errors___5 Error in Application of Statistical Methods or Data Analysis<br>6 errors___6 Error in Selection or Reporting of Analysis for Publication<br>7 errors___7 Concerns about Image(s) or Figure(s)<br>8 errors___8 Results Could Not Be Reproduced or Replicated<br>9 errors___9 Other<br>99 errors___99 Cannot tell

	268	[datacollection]<br><br>Show the field ONLY if:<br>[errors(1)] = '1'	Extract (related to data collection or capture)	notes
	269	[dataclean]<br><br>Show the field ONLY if:<br>[errors(2)] = '1'	Extract (related to data cleaning or preparation)	notes
	270	[datalost]<br><br>Show the field ONLY if:<br>[errors(3)] = '1'	Extract (data no longer available or lost)	notes
	271	[conduct]<br><br>Show the field ONLY if:<br>[errors(4)] = '1'	Extract (study conduct)	notes
	272	[method]<br><br>Show the field ONLY if:<br>[errors(5)] = '1'	Extract (related to data analysis)	notes
	273	[selection]<br><br>Show the field ONLY if:<br>[errors(6)] = '1'	Extract (related to selection of results)	notes
	274	[image]<br><br>Show the field ONLY if:<br>[errors(7)] = '1'	Extract (related to image)	notes
	275	[replication]<br><br>Show the field ONLY if:<br>[errors(8)] = '1'	Extract (related to replication or reproduction)	notes
	276	[otherreason]<br><br>Show the field ONLY if:<br>[errors(9)] = '1'	Extract (other)	notes
	277	[qualitative_review_complete]	Section Header: *Form Status*<br>Complete?	dropdown<br>0 Incomplete<br>1 Unverified<br>2 Complete
**Instrument: Qualitative Review 2 (qualitative_review_2)**				
	278	[qual_reviewer_v2]	Section Header: *Study Information: Title: [title] Journal: [journal] Country: [country] Authors: [author] Publication Date: [published] Retraction Date: [retracted] Retraction URL: [url] Publication DOI: [publication_doi] Retraction DOI: [retraction_doi] Reason List from Retraction Watch: [reason_list] Retraction Notice: [retraction_notice]*<br>Reviewer	radio, Required<br>4 Grace Bellinger<br>5 Oriana Fleming<br>0 Abigail Baldridge<br>1 Eric Whitely<br>2 Leah Welty<br>3 Luke Rasmussen
	279	[adequate_v2]	Does the retraction contain adequate information to perform a qualitative review?	yesno<br>1 Yes<br>0 No

	280	[errors_v2]<br><br>Show the field ONLY if:<br>[adequate_v2] = '1'	What types of errors are described in the retraction notice?	checkbox, Required<br>1 errors_v2___1 Data Collection, Capture, Unreliable, or Unverifiable Data<br>2 errors_v2___2 Data Cleaning, Preparation, or Variable Coding<br>3 errors_v2___3 Data No Longer Available or Lost<br>4 errors_v2___4 Error in Study Conduct, Protocol Violation, Ethics<br>5 errors_v2___5 Error in Application of Statistical Methods or Data Analysis<br>6 errors_v2___6 Error in Selection or Reporting of Analysis for Publication<br>7 errors_v2___7 Concerns about Image(s) or Figure(s)<br>8 errors_v2___8 Results Could Not Be Reproduced or Replicated<br>9 errors_v2___9 Other<br>99 errors_v2___99 Cannot tell
	281	[datacollection_v2]<br><br>Show the field ONLY if:<br>[errors_v2(1)] = '1'	Extract (related to data collection or capture)	notes
	282	[dataclean_v2]<br><br>Show the field ONLY if:<br>[errors_v2(2)] = '1'	Extract (related to data cleaning or preparation)	notes
	283	[datalost_v2]<br><br>Show the field ONLY if:<br>[errors_v2(3)] = '1'	Extract (data no longer available or lost)	notes
	284	[conduct_v2]<br><br>Show the field ONLY if:<br>[errors_v2(4)] = '1'	Extract (study conduct)	notes
	285	[method_v2]<br><br>Show the field ONLY if:<br>[errors_v2(5)] = '1'	Extract (related to data analysis)	notes
	286	[selection_v2]<br><br>Show the field ONLY if:<br>[errors_v2(6)] = '1'	Extract (related to selection of results)	notes
	287	[image_v2]<br><br>Show the field ONLY if:<br>[errors_v2(7)] = '1'	Extract (related to image)	notes
	288	[replication_v2]<br><br>Show the field ONLY if:<br>[errors_v2(8)] = '1'	Extract (related to replication or reproduction)	notes
	289	[otherreason_v2]<br><br>Show the field ONLY if:<br>[errors_v2(9)] = '1'	Extract (other)	notes
	290	[qualitative_review_2_complete]	Section Header: *Form Status*<br>Complete?	dropdown<br>0 Incomplete<br>1 Unverified<br>2 Complete

Instrument: Qualitative Review 3 (qualitative_review_3) **Enabled as survey**

	291	[qual_reviewer_v3]	Section Header: *Study Information: Title: [title] Journal: [journal] Country: [country] Authors: [author] Publication Date: [published] Retraction Date: [retracted] Retraction URL: [url] Publication DOI: [publication_doi] Retraction DOI: [retraction_doi] Reason List from Retraction Watch: [reason_list] Retraction Notice: [retraction_notice]*<br>Reviewer	radio, Required<br>4 Grace Bellinger<br>5 Oriana Fleming<br>0 Abigail Baldridge<br>1 Eric Whitely<br>2 Leah Welty<br>3 Luke Rasmussen
	292	[problem_get_acquire_data_v3]	Does the retraction notice indicate there was a problem with GETTING/ACQUIRING DATA? Examples (even if discovered by inspecting a figure): laboratory error, sample contamination, incorrect papers in meta-analysis, wrong cell types, incorrect data pulls from an EHR or other system, unreliable/concerns about data, error in data, or loss of data. What also counts: if they regenerate data and get a different answer, or if there is a problem with data storage (getting, saving, retaining).This also includes errors related to generating the raw data -- for example incorrect patient identification for a case, or an error pulling from the EMR or misinterpretation of diagnoses or tests in the data pull. Intentional manipulation of data (scientific misconduct) does NOT count for this category.	yesno, Required<br>1 Yes<br>0 No
	293	[problem_get_acquire_data_quote_v3]<br>Show the field ONLY if:<br>[problem_get_acquire_data_v3] = '1'	Extract (Getting/Acquiring Data)	notes
	294	[problem_prepare_analyze_data_v3]	Does the retraction notice indicate there was a problem with PREPARING OR ANALYZING DATA? Examples (even if discovered by inspecting a figure): data manipulation or preparation, data cleaning, data normalization, unit conversion, incorrect data merge, variable coding, statistical analysis, wrong standard errors, possibly unreliable/concerns about results as long as it's suggested that they're from data analysis and not benchtop or data generation problems. Intentional manipulation of data (scientific misconduct) does NOT count for this category.	yesno, Required<br>1 Yes<br>0 No
	295	[problem_prepare_analyze_data_quote_v3]<br>Show the field ONLY if:<br>[problem_prepare_analyze_data_v3] = '1'	Extract (Preparing/Analyzing Data)	notes
	296	[problem_any_v3]<br>Show the field ONLY if:<br>[problem_prepare_analyze_data_v3] = '0' and [problem_get_acquire_data_v3] = '0'	Does the retraction notice provide any other information about why the article was retracted? You should say "No" if the retraction notice text is empty, or states no reason whatsoever (e.g., it only states "The article has been retracted at the request of the authors")	yesno<br>1 Yes<br>0 No
	297	[problem_other_v3]<br>Show the field ONLY if:<br>[problem_any_v3] = '1'	Extract (Other Problem) Examples:General statements about "could not be replicated" that do not refer specifically to data or resultsQuestions about the integrity of the data (not about the process generating the data)Duplicate figures/article published in another context	notes
	298	[qualitative_review_3_complete]	Section Header: *Form Status*<br>Complete?	dropdown<br>0 Incomplete<br>1 Unverified<br>2 Complete

Instrument: **Qualitative Review 4** (qualitative_review_4) **Enabled as survey**

	299	[qual_reviewer_v4]	Section Header: *Study Information: Title: [title] Journal: [journal] Country: [country] Authors: [author] Publication Date: [published] Retraction Date: [retracted] Retraction URL: [url] Publication DOI: [publication_doi] Retraction DOI: [retraction_doi] Reason List from Retraction Watch: [reason_list] Retraction Notice: [retraction_notice]*<br>Reviewer	radio, Required<br>4 Grace Bellinger<br>5 Oriana Fleming<br>0 Abigail Baldridge<br>1 Eric Whitely<br>2 Leah Welty<br>3 Luke Rasmussen
	300	[problem_get_acquire_data_v4]	Does the retraction notice indicate there was a problem with GETTING/ACQUIRING DATA? Examples (even if discovered by inspecting a figure): laboratory error, sample contamination, incorrect papers in meta-analysis, wrong cell types, incorrect data pulls from an EHR or other system, unreliable/concerns about data, error in data, or loss of data. What also counts: if they regenerate data and get a different answer, or if there is a problem with data storage (getting, saving, retaining).This also includes errors related to generating the raw data -- for example incorrect patient identification for a case, or an error pulling from the EMR or misinterpretation of diagnoses or tests in the data pull. Intentional manipulation of data (scientific misconduct) does NOT count for this category.	yesno, Required<br>1 Yes<br>0 No
	301	[problem_get_acquire_data_quote_v4]<br>Show the field ONLY if:<br>[problem_get_acquire_data_v4] = '1'	Extract (Getting/Acquiring Data)	notes
	302	[problem_prepare_analyze_data_v4]	Does the retraction notice indicate there was a problem with PREPARING OR ANALYZING DATA? Examples (even if discovered by inspecting a figure): data manipulation or preparation, data cleaning, data normalization, unit conversion, incorrect data merge, variable coding, statistical analysis, wrong standard errors, possibly unreliable/concerns about results as long as it's suggested that they're from data analysis and not benchtop or data generation problems. Intentional manipulation of data (scientific misconduct) does NOT count for this category.	yesno, Required<br>1 Yes<br>0 No
	303	[problem_prepare_analyze_data_quote_v4]<br>Show the field ONLY if:<br>[problem_prepare_analyze_data_v4] = '1'	Extract (Preparing/Analyzing Data)	notes
	304	[problem_any_v4]<br>Show the field ONLY if:<br>[problem_prepare_analyze_data_v4] = '0' and [problem_get_acquire_data_v4] = '0'	Does the retraction notice provide any other information about why the article was retracted? You should say "No" if the retraction notice text is empty, or states no reason whatsoever (e.g., it only states "The article has been retracted at the request of the authors")	yesno<br>1 Yes<br>0 No
	305	[problem_other_v4]<br>Show the field ONLY if:<br>[problem_any_v4] = '1'	Extract (Other Problem) Examples:General statements about "could not be replicated" that do not refer specifically to data or resultsQuestions about the integrity of the data (not about the process generating the data)Duplicate figures/article published in another context	notes
	306	[qualitative_review_4_complete]	Section Header: *Form Status*<br>Complete?	dropdown<br>0 Incomplete<br>1 Unverified<br>2 Complete

Instrument: Consensus Review (consensus_review)

	307	[qual_reviewer_c]	Section Header: *Study Information: Title: [title] Journal: [journal] Country: [country] Authors: [author] Publication Date: [published] Retraction Date: [retracted] Retraction URL: [url] Publication DOI: [publication_doi] Retraction DOI: [retraction_doi] Reason List from Retraction Watch: [reason_list] Retraction Notice: [retraction_notice]*<br>Reviewer	radio, Required<br>6 Team
	308	[problem_get_acquire_data_c]	Does the retraction notice indicate there was a problem with GETTING/ACQUIRING DATA? Examples (even if discovered by inspecting a figure): laboratory error, sample contamination, incorrect papers in meta-analysis, wrong cell types, incorrect data pulls from an EHR or other system, unreliable/concerns about data, error in data, or loss of data. What also counts: if they regenerate data and get a different answer, or if there is a problem with data storage (getting, saving, retaining).This also includes errors related to generating the raw data -- for example incorrect patient identification for a case, or an error pulling from the EMR or misinterpretation of diagnoses or tests in the data pull. Intentional manipulation of data (scientific misconduct) does NOT count for this category.	yesno, Required<br>1 Yes<br>0 No
	309	[problem_get_acquire_data_quote_c]<br>Show the field ONLY if: [problem_get_acquire_data_c] = '1'	Extract (Getting/Acquiring Data)	notes
	310	[problem_prepare_analyze_data_c]	Does the retraction notice indicate there was a problem with PREPARING OR ANALYZING DATA? Examples (even if discovered by inspecting a figure): data manipulation or preparation, data cleaning, data normalization, unit conversion, incorrect data merge, variable coding, statistical analysis, wrong standard errors, possibly unreliable/concerns about results as long as it's suggested that they're from data analysis and not benchtop or data generation problems. Intentional manipulation of data (scientific misconduct) does NOT count for this category.	yesno, Required<br>1 Yes<br>0 No
	311	[problem_prepare_analyze_data_quote_c]<br>Show the field ONLY if: [problem_prepare_analyze_data_c] = '1'	Extract (Preparing/Analyzing Data)	notes
	312	[problem_any_c]<br>Show the field ONLY if: [problem_prepare_analyze_data_c] = '0' and [problem_get_acquire_data_c] = '0'	Does the retraction notice provide any other information about why the article was retracted? You should say "No" if the retraction notice text is empty, or states no reason whatsoever (e.g., it only states "The article has been retracted at the request of the authors")	yesno<br>1 Yes<br>0 No
	313	[problem_other_c]<br>Show the field ONLY if: [problem_any_c] = '1'	Extract (Other Problem) Examples:General statements about "could not be replicated" that do not refer specifically to data or resultsQuestions about the integrity of the data (not about the process generating the data)Duplicate figures/article published in another context	notes
	314	[consensus_review_complete]	Section Header: *Form Status*<br>Complete?	dropdown<br>0 Incomplete<br>1 Unverified<br>2 Complete

Abstract Review

Record ID	|__|__|__|__|__|__|	record_id
Date of Abstract Review	|__|__|/|__|__|/|2|0|__|__|	abstract_review_date
Reviewer	☐ Abigail Baldridge (0)<br>☐ Eric Whitley (1)<br>☐ Leah Welty (2)<br>☐ Luke Rasmussen (3)	abstract_reviewer
**Inclusion/Exclusion Criteria:**		
Is the article written in the English language?	☐ Yes (1)<br>☐ No (0) →	english
→ If no, then the article is not eligible		
Is the article about human subjects research?	☐ Yes (1)<br>☐ No (0)	human_research
If the article is not about human subjects research, is it applicable to the human translational research pipeline?	☐ Yes (1)<br>☐ No (0) →	translational
→ If no, the article is not eligible		
Is the article eligible for full text review?	☐ Yes (1)<br>☐ No (0)	eligibility
End of Form		

Qualitative Review

Record ID	|__|__|__|__|__|__|	record_id
Reviewer	☐ Grace Bellinger (4)<br>☐ Oriana Fleming (5)<br>☐ Abigail Baldridge (0)<br>☐ Eric Whitley (1)<br>☐ Leah Welty (2)<br>☐ Luke Rasmussen (3)	qual_reviewer
Does the retraction contain adequate information to perform a qualitative review?	☐ Yes (1)<br>☐ No (0) →	adequate
→ If no, then end the review		
Who is specifically named in the retraction statement as retracting the article?<br><br>Select all that apply	☐ Author(s) (1)<br>☐ Ambiguous Editor(s)/Journal/Publisher (2)<br>☐ Editor(s) (3)<br>☐ Journal (4)<br>☐ Publisher (5)<br>☐ Other (6) →<br>☐ Not Stated (7)	retractor
→ If other, please list		other_retractor
Who initiated the investigation or discovered that the retraction should occur?<br><br>Select all that apply	☐ Author(s) (1)<br>☐ Editor(s)/Journal/Publisher (2)<br>☐ Letter to the Editor (3)<br>☐ External Investigation (4)<br>☐ Readers (5)<br>☐ Unnamed Entity (6)<br>☐ Other (7) →<br>☐ Not Stated (8)	initiator
→ If other, please list		other_initiator
Were any of the authors involved in the retraction process?	☐ Yes (1) →<br>☐ No, the authors were unresponsive (2)<br>☐ No / Not stated (3)	authors_responded

→ Do any of the authors disagree with the retraction?	☐ Yes (1)<br>☐ No (2)<br>☐ Not stated (3)	agree
What types of errors are described in the retraction notice?<br><br>Select all that apply	☐ Data Collection, Capture, Unreliable, or Unverifiable Data (1)<br>☐ Data Cleaning, Preparation, or Variable Coding (2)<br>☐ Data No Longer Available or Lost (3)<br>☐ Error in Study Conduct, Protocol Violation, Ethics (4)<br>☐ Error in Application of Statistical Methods or Data Analysis (5)<br>☐ Error in Selection or Reporting of Analysis for Publication (6)<br>☐ Concerns about Image(s) or Figure(s) (7)<br>☐ Results Could Not Be Reproduced or Replicated (8)<br>☐ Other (9)<br>☐ Cannot tell (99)	errors
For each error noted above, copy and paste the excerpt(s) relating to that error		
Extract (related to data collection or capture)		datacollection
Extract (related to data cleaning or preparation)		dataclean
Extract (data no longer available or lost)		datalost
Extract (study conduct)		conduct
Extract (related to data analysis)		method
Extract (related to selection of results)		selection

Extract (related to image)		Image
Extract (related to replication or reproduction)		Replication
Extract (other)		otherreason
Does the retraction notice indicate there was a problem with GETTING/ACQUIRING DATA?<br><br>Examples (even if discovered by inspecting a figure): laboratory error, sample contamination, incorrect papers in meta-analysis, wrong cell types, incorrect data pulls from an EHR or other system, unreliable/concerns about data, error in data, or loss of data.<br><br>What also counts: if they regenerate data and get a different answer, or if there is a problem with data storage (getting, saving, retaining).<br>This also includes errors related to generating the raw data -- for example incorrect patient identification for a case, or an error pulling from the EMR or misinterpretation of diagnoses or tests in the data pull.<br><br>Intentional manipulation of data (scientific misconduct) does NOT count for this category.	☐ Yes (1) →<br>☐ No (0)	problem_get_acquire_data
→ If yes, Extract		problem_get_acquire_data_quote
Does the retraction notice indicate there was a problem with PREPARING OR ANALYZING DATA?<br><br>Examples (even if discovered by inspecting a figure): data manipulation or preparation, data cleaning, data normalization, unit conversion, incorrect data merge, variable coding, statistical analysis, wrong standard errors, possibly unreliable/concerns about results as long as it's	☐ Yes (1) →<br>☐ No (0)	problem_prepare_analyze_data

suggested that they're from data analysis and not benchtop or data generation problems.

Intentional manipulation of data (scientific misconduct) does NOT count for this category.

→ If yes, Extract — problem_prepare_analyze_data_quote

Does the retraction notice provide any other information about why the article was retracted? — ☐ Yes (1) → ☐ No (0) — problem_any

You should say "No" if the retraction notice text is empty, or states no reason whatsoever (e.g., it only states "The article has been retracted at the request of the authors")

→ If yes, Extract — problem_other

End of Form

Full Text Review

Record ID	|__|__|__|__|__|__|	record_id
Date of Full Text Review	|__|__|/|__|__|/|2|0|__|__|	full_text_review_date
Reviewer	☐ Abigail Baldridge (0)<br>☐ Eric Whitley (1)<br>☐ Leah Welty (2)<br>☐ Luke Rasmussen (3)<br>☐ Grace Bellinger (4)<br>☐ Oriana Fleming (5)	full_text_reviewer
**Author Information:**		
Is one or more of the authors a consortia?	☐ Yes (1)<br>☐ No (0)	consortia
Count the total Number of Individual Authors	|__|__|__|__|__|__|	author_num
**Retracted Article:**		
Copy and Paste the Verbatim Retraction Notice Here:		retraction_notice
Notes about the article:		notes
Is the full text of the retracted article available?	☐ Yes (1)<br>☐ No (0) →	human_research
→ If no, the full text review does not need to be completed. You may exit the survey.		
Please upload a pdf of the retracted article		file_upload
For all remaining questions, use ONLY the article itself, or its supplemental materials. If information are not included in the article or supplemental materials, these fields can be left blank or as "N/A".		
**Author Roles and Qualifications**		
Is there an attribution statement?	☐ Yes (1) →<br>☐ No (0)	attribauthor
→ Copy and paste the attribution statement here		attrib_statement

**Data Collection**		
What is the primary type of data used?	☐ De Novo Collected (0)<br>☐ Previously Collected for Research Purposes (1) →<br>☐ Previously Collected not Necessarily for Research (2) →<br>☐ Undefined (3)<br>☐ Other (4) →	data_type
→ For previously collected data, how was the data obtained for this publication?<br><br>Copy and paste all relevant statements, using a carriage return between separate statements.		data_source
→ List the Other Type of Data		other_data
Where was the primary data collected for the publication?	☐ Entered into Editable File (e.g. Excel, CSV, Tab Delimited) (0)<br>☐ Research Study Software (e.g. REDCap, CTMS) (1)<br>☐ Database Program (e.g. ACCESS, SQL) (2)<br>☐ Undefined (3)<br>☐ Other (4) →	data_storage
→ List the Other Data Collection Method/Location		other_data_storage
**Data Availability**		
Is the data used within this publication available?<br><br>Note: if it came from a publically available dataset, then yes.	☐ Publicly available (0)<br>☐ Available upon request (1)<br>☐ Unknown (2)	data_availability
**Methods**		
Is this a Systematic Review or Meta Analysis?	☐ Yes (1)	sys_review

Question	Response	Variable
	☐ No (0) →	
→ Is this Human Subjects research?	☐ Yes (1) →<br>☐ No (0)	human_subj
→ What was the human subjects design?	☐ Human: Clinical Subjects Trial (0)<br>☐ Human: Observational Study (1) →<br>☐ Benchtop (e.g. Cell lines, tissue) (2)<br>☐ Undefined (99)	study_design
→ What type of observational study was performed?	☐ Case-Control (0)<br>☐ Cross Sectional (1)<br>☐ Longitudinal / Repeated Measures (2)<br>☐ Case Report (3)<br>☐ Undefined (4)	observational
→ What was the temporality of the observational study?	☐ Retrospective (0)<br>☐ Prospective (1)<br>☐ Both (2)<br>☐ Undefined (3)<br>☐ NA (For Case Reports or Cross Sectional Studies) (4)	temporality
→ Were live animals used in this research study?	☐ Yes (1)<br>☐ No (0)	animal
Does the article state that a statistical analysis plan was pre-specified?	☐ Yes, publicly available (1)<br>☐ Yes, stated as pre-specified but not publicly available (2)<br>☐ Not explicitly stated (3)	sap
What software was used for analysis?	☐ Excel / Spreadsheet / Google Sheets (8)<br>☐ GraphPad PRISM (5)<br>☐ JMP (9)<br>☐ KaleidaGraph (10)<br>☐ Minitab (11)<br>☐ MPlus (13)<br>☐ Power BI (7)<br>☐ Python (3)	software

Full Text Review

	☐ R / R Studio (1)<br>☐ SAS (0)<br>☐ SPSS / PASW / PASW18 (4)<br>☐ Stata (2)<br>☐ Statistica (12)<br>☐ Tableau (6)<br>☐ Undefined (999)<br>☐ Other (777) →	
→ List the other software		other_software
Is there any additional information about the use of reproducible research methods?	☐ Yes (1) →<br>☐ No (0)	reproducible_methods
→ Copy and paste the verbatim language about reproducible methods here:		reproducible_statement

End of Form
